# A Comprehensive Analysis of Population Differences in *LRRK2* Variant Distribution in Parkinson's Disease

**DOI:** 10.3389/fnagi.2019.00013

**Published:** 2019-01-30

**Authors:** Li Shu, Yuan Zhang, Qiying Sun, Hongxu Pan, Beisha Tang

**Affiliations:** ^1^Department of Neurology, Xiangya Hospital, Central South University, Changsha, China; ^2^Department of Geriatrics, Xiangya Hospital, Central South University, Changsha, China; ^3^National Clinical Research Center for Geriatric Disorders, Changsha, China; ^4^Key Laboratory of Hunan Province in Neurodegenerative Disorders, Central South University, Changsha, China; ^5^Parkinson's Disease Center of Beijing Institute for Brain Disorders, Beijing, China

**Keywords:** Parkinson's disease, *LRRK2*, Asian, European, variant

## Abstract

**Background:** *LRRK2* variants have been demonstrated to have distinct distributions in different populations. However, researchers have thus far chosen to focus on relatively few variants, such as R1628P, G2019S, and G2385R. We therefore investigated the relationship between common *LRRK2* variants and PD risk in various populations.

**Methods:** Using a set of strict inclusion criteria, six databases were searched, resulting in the selection of 94 articles covering 49,299 cases and 47,319 controls for final pooled analysis and frequency analysis. Subgroup analysis were done for Africans, European/West Asians, Hispanics, East Asians, and mixed populations. Statistical analysis was carried out using the Mantel-Haenszel approach to determine the relationship between common *LRRK2* variants and PD risk, with the significance level set at *p* < 0.05.

**Results:** In the absence of obvious heterogeneities and publication biases among the included studies, we concluded that A419V, R1441C/G/H, R1628P, G2019S, and G2385R were associated with increased PD risk (*p*: 0.001, 0.0004, < 0.00001, < 0.00001, and < 0.00001, respectively), while R1398H was associated with decreased risk (*p*: < 0.00001). In East Asian populations, A419V, R1628P, and G2385R increased risk (*p*: 0.001, < 0.00001, < 0.00001), while R1398H had the opposite effect (*p*: 0.0005). G2019S increased PD risk in both European/West Asian and mixed populations (*p*: < 0.00001, < 0.00001), while R1441C/G/H increased risk in European/West Asian populations only (*p*: 0.0004).

**Conclusions:** We demonstrated that *LRRK2* variant distribution is different among various populations, which should inform decisions regarding the development of future genetic screening strategies.

## Introduction

Parkinson's disease (PD) is one of the most common neurodegenerative diseases, affecting ~2% of people over the age of 60 years, and the most common cause of movement disorders, including bradykinesia, resting tremor, rigidity, and postural instability or gait difficulty. Non-motor symptoms, such as depression, olfactory dysfunction, and constipation are also common in PD (Schapira, 2006). The pathological hallmark of PD is Lewy Body aggregation in neurons and the loss of dopamine neurons in the substantia nigra compacta and corpus striatum.

The pathogenesis of PD is as yet unclear, but genetic and environmental factors, as well as aging are thought to contribute to PD risk. Since the discovery of the *PARK1* locus, further autosomal-dominant or -recessive disease genes have been identified (Paisan-Ruiz, 2009). The most common among the former is the leucine-rich repeat kinase 2 (*LRRK2*) gene, which encodes for a protein containing armadillo (ARM), Ras of complex proteins (ROC), C-terminal of ROC (COR), mitogen-activated protein kinase kinase kinase (MAPKKK), and WD40 domains, in addition to others (Kruger, 2008).

To date, nearly a hundred *LRRK2* variants have been identified. Of these, G2019S, R1628P, and G2385R have traditionally received much of the attention, and there have already been a number of meta-analyses on the role of these variants in PD risk in people of different ethnicities (Xie et al., 2014; Liu et al., 2016; Zhang et al., 2016; Zhao and Kong, 2016). These variants each possess distinct geographical distributions. G2019S, which is the most frequently-occurring variant, accounts for 3–6% of familial PD cases among patients in European populations (Guo et al., 2006) and nearly 14% of Ashkenazi Jews (AJ) (Luzon-Toro et al., 2007), whereas G2385R and R1628P were found to be more common among East Asian PD patients (Fu et al., 2013).

If strict selection criteria and quality control methods are employed, meta-analysis can be an immensely powerful tool, allowing researchers to pool data from original studies and effectively expanding the sample size (Haines et al., 2008) to provide unbiased evidence with far-reaching clinical implications (Wolf, 2015). In the case of genetic analyses in particular, where original studies are inevitably limited by the diversity of their subject pool, and by their limited sample sizes a meta-analytical approach can be invaluable in providing convincing evidence for the effects of a specific gene on disease risks. In the recent crop of articles, researchers have begun to shift their focus to the other less well-known *LRRK2* variants. Given that others have not yet done so, we decided in this study to perform a complete analysis of all relevant original association studies relating to the *LRRK2* variants that have been identified thus far.

## Methods

### Literature Search

The English-language Medline and Embase databases, as well as the Chinese-language Wanfang, CNKI, and VIP databases were searched manually on Nov 29, 2018, using the keywords “parkinson^*^,” “PD,” “*LRRK2*,” “PARK8,” “polymorphisms,” “SNP,” “gene,” “variant,” and “mutation.” Overlapping articles among databases were deleted in EndNote.

### Selection Criteria

The PICOS (participants, interventions, controls, outcomes, and study types) approach was used to define inclusion criteria. For this study, “participants” were patients diagnosed with PD according to accepted standards, such as the UK PD Society Brain Bank Clinical Diagnostic Criteria (Hughes et al., 1992) and other widely accepted criteria (Calne et al., 1992; Bower et al., 1999; Gelb et al., 1999); “Interventions” consisted of gDNA analysis performed using accepted methods based on PCR; “Controls” were people without PD or related diseases (movement disorders, neurodegenerative diseases etc.); We accepted the definition of PD patients or controls by the authors of the original articles if there were no description of the criteria; “Outcomes” were complete data (complete number of patients or controls carrying either homozygous or heterozygous polymorphisms of *LRRK2*) and at least four original articles reported on the same variant, and “study types” consisted of original case-control studies, cohort studies (Supplementary Table 1).

### Data Extraction

Complete data, including containing the name of the first author, the publication year of the study, subject ethnicity, the country in which the study was performed, the gene and gene variants analyzed, the number of cases and controls and subject genotypes (both homozygotes and heterozygotes) were extracted from all selected original studies, and are detailed in the Table 1, Supplementary Table 2. Pooled analysis was performed in cases where sufficient data was provided to allow for the calculation of odds ratios (OR) and 95% confidence intervals (CI). If studies had enough data to calculated frequency of variants, we included the articles in the frequency analysis. Newcastle-Ottawa Scale (NOS) was used to perform quality control on all included studies. Data extraction was performed by Li S and Yuan Z, in consultation with Qiying S. Results relating to R1628P were provided by ZY, a co-author on this study, and were based on (Zhang et al., 2017).

**Table 1:** The characteristics of all included publications.

**References**	**Ethnicity**	**Country**	**Researched variants**	**PD**	**Controls**	**NOS**
Tan et al., 2005	East Asians	Singapore	G2019S	675	325	8
Aasly et al., 2005	European/West Asians	Norway	G2019S	435	519	7
Berg et al., 2005	European/West Asians	Germany	G2019S, R793M	337	1,200	8
Bras et al., 2005	European/West Asians	Portugal	G2019S	124	126	6
Gilks et al., 2005	European/West Asians	UK	G2019S	482	345	6
Goldwurm et al., 2005	European/West Asians	Italy	G2019S, R1441C	629	440	8
Hernandez et al., 2005	European/West Asians	America	G2019S	719	2,680	8
Mata et al., 2005	European/West Asians	Spain	R1441G	225	100	7
Deng et al., 2005	Mixed	North American	G2019S	326	130	7
Farrer et al., 2005	Mixed	America	G2019S, R793M	786	278	7
Kachergus et al., 2005	Mixed	Europe and North America	G2019S	1,054	2,260	8
Nichols et al., 2005	Mixed	North America	G2019S	767	965	7
Di Fonzo et al., 2006	East Asians	Taiwan	A419V, G2385R, P755L	592	344	8
Funayama et al., 2007	East Asians	Japan	G2385R	448	457	8
Fung et al., 2006a	East Asians	Taiwan	G2385R	305	176	7
Fung et al., 2006b	East Asians	Taiwan	G2019S	343	213	7
Punia et al., 2006	East Asians	India	G2019S	800	212	7
Wu et al., 2006	East Asians	China	P755L	598	765	8
Infante et al., 2006	European/West Asians	Spain	G2019S	105	310	7
Carmine Belin et al., 2006	European/West Asians	Sweden	G2019S	284	305	7
Marongiu et al., 2006	European/West Asians	Italy	G2019S	1,072	300	8
Mata et al., 2006	European/West Asians	Spain	G2019S	225	100	7
Pchelina et al., 2006	European/West Asians	Russia	G2019S	208	161	7
Schlitter et al., 2006	European/West Asians	Germany	G2019S	120	336	7
Williams-Gray et al., 2006	European/West Asians	UK	G2019S	519	887	8
Clark et al., 2006	Mixed	America	G2019S	504	314	8
Deng et al., 2006	Mixed	North America	G2019S	496	220	7
Kay et al., 2006	Mixed	America	G2019S	1,518	1,733	9
Ozelius et al., 2006	Mixed	America	G2019S	120	317	7
Civitelli et al., 2007	European/West Asians	Italy	G2019S	488	180	7
Cossu et al., 2007	European/West Asians	Italy	G2019S	98	55	6
Farrer et al., 2007	East Asians	Taiwan	G2385R	410	335	8
Li et al., 2007	East Asians	China	G2385R	235	214	7
Orr-Urtreger et al., 2007	European/West Asians	Israel	G2019S	472	1,802	8
Tan et al., 2007a	East Asians	Singapore	G2385R	495	494	8
Tan et al., 2007b	East Asians	Singapore	G2385R	166	306	7
Toft et al., 2007	European/West Asians	Norway	R793M	433	587	7
Xiromerisiou et al., 2007	European/West Asians	Greece	G2019S	290	235	7
Aguiar Pde et al., 2008	Hispanics	Brazil	G2019S	72	72	6
Hulihan et al., 2008	African	Tunis	G2019S	238	371	7
An et al., 2008	East Asians	China	G2385R	600	334	8
Choi et al., 2008	East Asians	Korea	G2385R	72	100	6
Chan et al., 2008	East Asians	Hong Kong	G2385R	82	31	6
Lu et al., 2008	East Asians	China	R1628P	834	543	8
Ross et al., 2008	East Asians	Taiwan and Singapore	R1628P	1,079	907	9
Tan et al., 2008a	East Asians	Singapore	P755L	204	235	7
Tan et al., 2008b	East Asians	Singapore	R1628P	192	265	7
Tan et al., 2008c	East Asians	Singapore	R1628P	246	243	7
Tomiyama et al., 2008	East Asians	Japan	P755L	501	583	8
Bras et al., 2008	European/West Asians	Portugal	G2019S	132	126	7
Floris et al., 2009	European/West Asians	Italy	G2019S, R1441C	356	208	7
Patra et al., 2009	European/West Asians	America	G2019S	575	186	7
Yu et al., 2009	East Asians	China	R1628P	328	300	7
Zabetian et al., 2009	East Asians	Japan	G2019S, G2385R	631	320	8
Zhang et al., 2009	East Asians	China	R1628P	600	459	8
Gorostidi et al., 2009	European/West Asians	Spain	G2019S, R1441G	418	138	7
Hassin-Baer et al., 2009	European/West Asians	Israel	G2019S	242	900	7
Lesage et al., 2006	Mixed	Europe and North Africa	G2019S	226	174	7
Jasinska-Myga et al., 2010	African	Tunis	G2019S	240	372	7
Chen et al., 2011	East Asians	China	R1398H	430	452	7
Kim et al., 2010	East Asians	Korea	G2385R, R1628P	923	422	8
Miyake et al., 2010	East Asians	Japan	G2385R	229	358	7
Yescas et al., 2010	Hispanics	Mexico	G2019S	319	200	7
Hu et al., 2011	East Asians	China	G2019S	221	120	7
Lin et al., 2011	East Asians	Taiwan	G2385R, R1628P, S1647T	453	291	7
Pulkes et al., 2011	East Asians	Thailand	G2385R, R1628P	154	156	6
Yao et al., 2011	East Asians	China	P755L	401	398	8
Zheng et al., 2011	East Asians	China	S1647T	406	412	7
Li et al., 2012	East Asians	China	A419V	729	585	8
Vishwanathan Padmaja et al., 2012	East Asians	India	G2019S	140	201	7
Wang et al., 2012	East Asians	China	R1628P	2,013	1,971	9
Yan et al., 2012a	East Asians	China	G2385R	354	340	8
Yan et al., 2012b	East Asians	China	S1647T	354	160	6
Zhou et al., 2012	East Asians	China	G2385R, R1628P	202	212	7
Cai et al., 2013	East Asians	China	G2385R, R1628P	510	550	8
Fu et al., 2013	East Asians	China	G2385R, R1628P	446	403	7
Gopalai et al., 2013	East Asians	Malaysia	A419V	404	424	8
Li B. et al., 2013	East Asians	China	G2385R	197	202	6
Li Z. et al., 2013	East Asians	China	G2385R	237	190	6
Ma et al., 2013	East Asians	China	G2385R	237	190	7
Wu et al., 2013	East Asians	Taiwan	G2385R, R1398H, R1628P, S1647T	573	503	9
Wu-Chou et al., 2013	East Asians	Taiwan	G2385R, P755L, R1398H, R1628P	941	618	8
Chung et al., 2013	East Asians	Korean	G2385R	1,032	1,201	8
Dan et al., 2014	East Asians	China	G2385R	561	556	8
Guo et al., 2015	East Asians	China	G2385R, R1628P	1,020	1,031	8
Pulkes et al., 2014	East Asians	Thailand	R1628P	485	480	7
Chien et al., 2014	Hispanics	Multi-countries	G2019S	100	100	8
Heckman et al., 2014	East Asians	Multi-countries	R1398H	1,345	938	9
Heckman et al., 2014	European/West Asians	Multi-countries	R1398H	5,894	4,282	9
Li K. et al., 2015	East Asians	China	A419V	500	574	7
Li X.X. et al., 2015	East Asians	China	G2019S	312	360	7
Duque et al., 2015	Hispanics	Colombia	G2019S	154	162	6
Bandres-Ciga et al., 2016	European/West Asians	Spain	G2019S, R793M	240	192	7
Landoulsi et al., 2017	African	Tunis	G2019S	250	218	7
Emelyanov et al., 2018	European/West Asians	Russia	G2019S	762	400	8

*PD, Parkinson's disease*.

### Statistical Analysis

Statistical analysis was performed using Revman 5.3 software. The pooled analyses were conducted if there were at least four original studies. Meta-analyses were conducted on total populations and subgroup analyses by ethnicity (Africans, European/West Asians, Hispanics, East Asians, mixed:composed of at least two different groups) (Risch et al., 2002; Zhang et al., 2018). In cases where the Q statistic *P* > 0.1 and *I*^2^ statistic ≤ 50%, a fixed-effect model was used, otherwise, a random-effect model was applied for pooled analysis instead. All pooled results were graphed using forest plots and publication biases were showed using funnel plots. Subgroup analysis using the Mantel-Haenszel statistical method was performed to determine how the common *LRRK2* variants affect PD risk and the level of significance was set at *p* < 0.05. Sensitivity analysis was performed by sequentially deleting each included article, and observing how the pooled OR and 95%CI was affected by their removal. Genotype frequency (GF) and minor allele frequency (MAF) were calculated of each *LRRK2* variants in our analyses.

## Results

**Figure 1:**
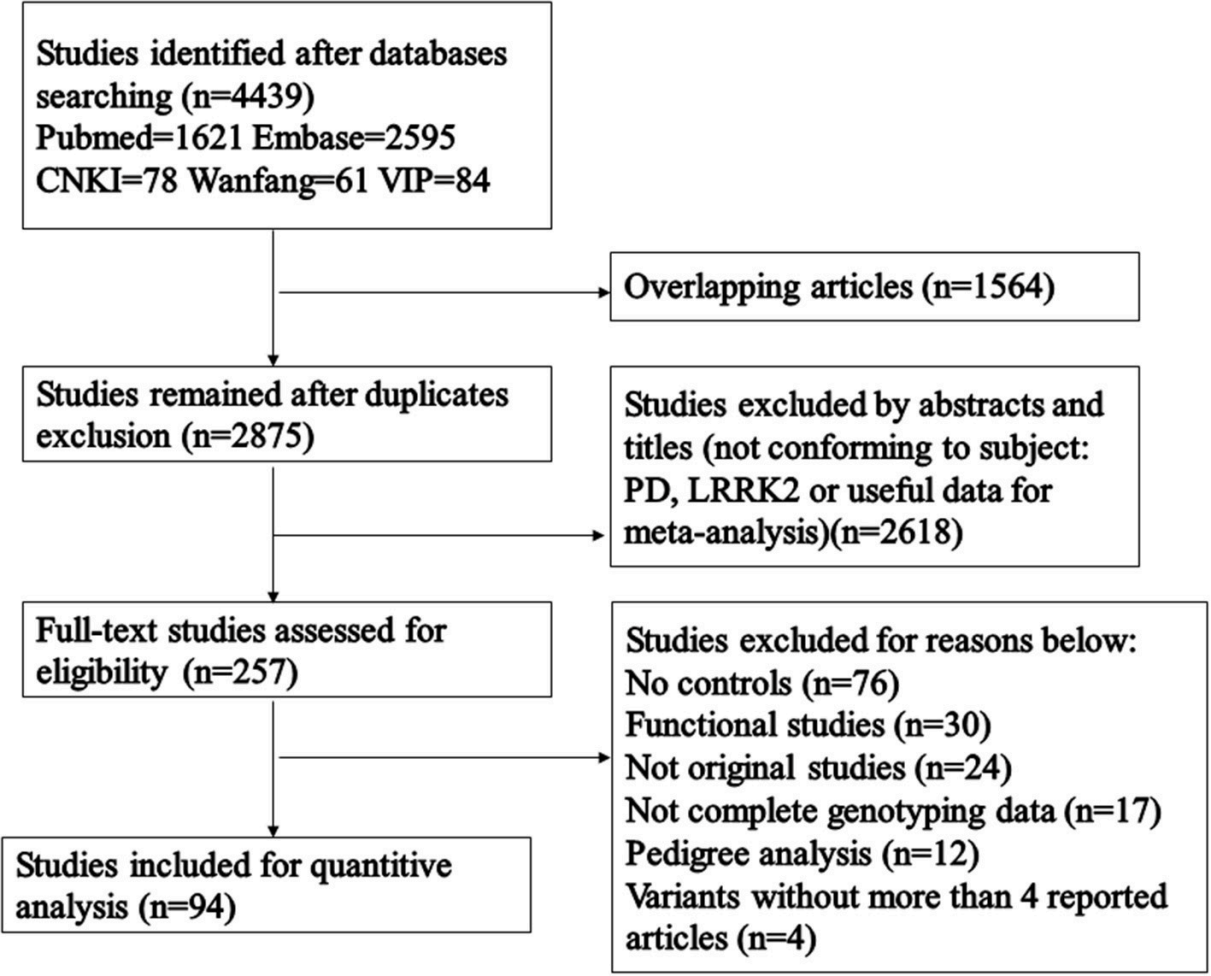
Flowchart illustrating the literature screening process.

**Figure 2:**
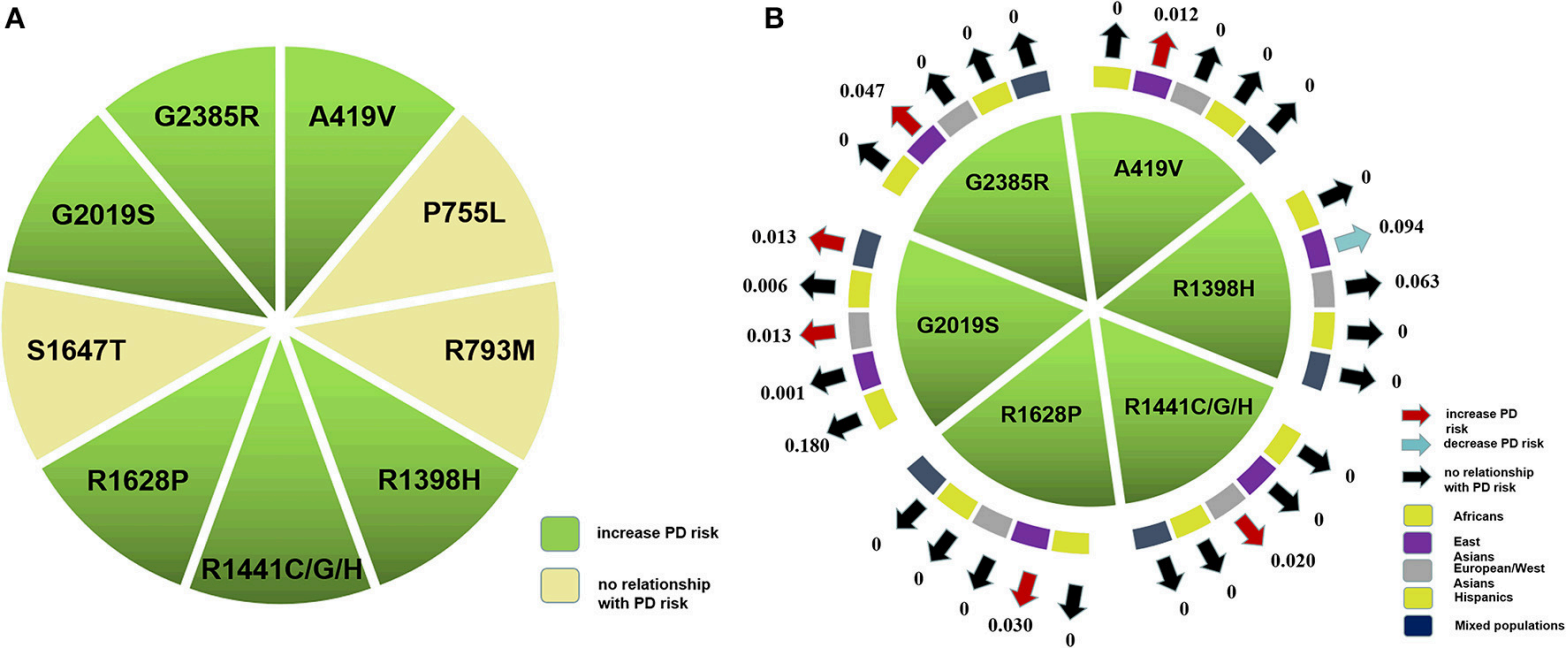
Nine *LRRK2* variants were included in the meta-analysis. **(A)** Six variants which had statistical differences in our pooled analysis. **(B)** Red, blue, and black arrows indicate that the associated variant increases, decreases, or has no bearing on PD risk, respectively. The arrow pointed to MAF of a specific variant.

A total of 4,439 articles were included following our initial database search (Figure 1). 2,875 articles remained after repeated articles had been eliminated, and a further 2,618 articles were excluded after we had manually reviewed their titles and abstracts. Among the 257 articles that received a full-text review, 163 were excluded due to no controls, functional studies, not original studies, not complete genotyping data, pedigree analysis and articles studying variants with no more than 4 reported articles. Eventually, 94 relevant articles were included in the final analysis, covering 49,299 cases and 47,319 control subjects (Table 1). As shown in Table 1, all included articles were of high quality. Subgroup analysis was performed for each of the major ethnic groups (Africans, European/West Asians, Hispanics, East Asians, mixed: composed of at least two different groups). Results of the pooled analysis were graphed using forest plots (Supplementary Figure 1), and variant frequencies in PD patients of different ethnicities were further calculated (Figure 2). All analysis was performed using a fixed-effect model due to there being relatively little heterogeneity among the included studies.

### Comprehensive Analysis of *LRRK2* Variants in Different Ethnicities

Based on our comprehensive analysis of the common *LRRK2* variants, we concluded that A419V, R1398H, R1441C/G/H, R1628P, G2019S, and G2385R were associated with greater PD risk (*p*: 0.001, 0.0004, < 0.00001, < 0.00001, and < 0.00001, respectively), while R1398H was associated with decreased PD risk (< 0.00001; Figure 2). Of the high-risk variants, G2019S posed the greatest degree of risk, followed by R1441C/G/H, A419V, G2385R, and R1628P in descending order, as demonstrated by their OR values, which ranged from 13.16 to 1.83 (Table 2).

**Table 2:** Relationship between common *LRRK2* and overall PD risk and for populations of specific ethnicities.

**Variants**	**Total population**	**Africans**	**East asians**	**European/west asians**	**Hispanics**	**Mixed**
A419V	4 (2,225/1,927)	0 (0/0)	4 (2,225/1,927)	0 (0/0)	0 (0/0)	0 (0/0)
	**2.45 [1.43, 4.20]**	NA	**2.45 [1.43, 4.20]**	NA	NA	NA
	**0.001**	NA	**0.001**	NA	NA	NA
P755L	6 (2,808/2,764)	0 (0/0)	6 (2,808/2,764)	0 (0/0)	0 (0/0)	0 (0/0)
	1.24 [0.97, 1.59]	NA	1.24 [0.97, 1.59]	NA	NA	NA
	0.09	NA	0.09	NA	NA	NA
R793M	4 (1,796/2,257)	0 (0/0)	0 (0/0)	3 (1,010/1,979)	0 (0/0)	1 (786/278)
	0.99 [0.30, 3.31]	NA	NA	NA	NA	NA
	0.99	NA	NA	NA	NA	NA
R1398H	5 (8,761/6,609)	0 (0/0)	4 (2,867/2,327)	1 (5,894/4,282)	0 (0/0)	0 (0/0)
	**0.81 [0.75, 0.89]**	NA	**0.78 [0.68, 0.90]**	NA	NA	NA
	**< 0.00001**	NA	**0.0005**	NA	NA	NA
R1441C/G/H	4 (1,599/711)	0 (0/0)	0 (0/0)	4 (1,599/711)	0 (0/0)	0 (0/0)
	**12.75 [3.11, 52.27]**	NA	NA	**12.75 [3.11, 52.27]**	NA	NA
	**0.0004**	NA	NA	**0.0004**	NA	NA
R1628P (Zhang et al., 2017)	16 (9,275/8,114)	0 (0/0)	16 (9,275/8,114)	0 (0/0)	0 (0/0)	0 (0/0)
	**1.83 [1.57, 2.13]**	NA	**1.83 [1.57, 2.13]**	NA	NA	NA
	**< 0.00001**	NA	**< 0.00001**	NA	NA	NA
S1647T	4 (1,786/1,366)	0 (0/0)	4 (1,786/1,366)	0 (0/0)	0 (0/0)	0 (0/0)
	1.05 [0.91, 1.21]	NA	1.05 [0.91, 1.21]	NA	NA	NA
	0.54	NA	0.54	NA	NA	NA
G2019S	47 (19,624/21,642)	3 (728/961)	7 (3,122/1,751)	24 (9,332/12,005)	3 (545/434)	10 (5,897/6,491)
	**13.16 [10.16, 17.04]**	NA	1.59 [0.18, 14.05]	**8.71 [6.12, 12.38]**	NA	**18.12 [9.26, 35.44]**
	**< 0.00001**	NA	0.67	**< 0.00001**	NA	**< 0.00001**
G2385R	27 (12,121/11,684)	0 (0/0)	27 (12,121/11,684)	0 (0/0)	0 (0/0)	0 (0/0)
	**2.27 [2.03, 2.53]**	NA	**2.27 [2.03, 2.53]**	NA	NA	NA
	**< 0.00001**	NA	**< 0.00001**	NA	NA	NA

*The number of articles (cases/controls), OR [95%CI], and p-value are shown. OR and 95%CI values shown in bold correspond to statistically significant results*.

By ethnicity, our analysis indicated that A419V, R1628P, and G2385R were associated with increased PD risk in East Asian populations (*p*: 0.001, < 0.00001, and < 0.00001), while R1398H had the opposite effect (*p*: 0.0005). The G2019S variant was found to increase PD risk in European/West Asian and mixed populations (*p*: < 0.00001 and < 0.00001), while R1441C/G/H increased risk for European/West Asians only (*p*: 0.0004; Table 2).

### *LRRK2* Variant Frequency in PD Patients and Control Individuals of Different Ethnicities

Among the *LRRK2* variants which were of statistical significance in previous meta-analyses, the most frequently-occurring *LRRK2* variants in PD patients were, in descending order, R1398H, G2385R, R1628P, and A419V, which had MAF ranging from 0.094 to 0.012 in East Asian populations (Figure 2; Supplementary Table 3). In European/West Asians, the MAF of the high-risk variants G2019S and R1441C/G/H were 0.013 and 0.020, respectively (Figure 2; Supplementary Table 3), and G2019S occurred within mixed populations at a total frequency of 0.013. Further, we found that A419V, R1628P, and G2385R appeared to be specific for Asian populations, while R1441C/G/H were European/West Asians-specific. Even though the total genotype frequency for other variants, such as S1647T and P755L were higher compared to those of G2385R or G2019S, a significant difference in their distribution between cases and control subjects was not apparent.

### Sensitivity Analysis and Publication Bias

After sequentially deleting each included article, the pooled OR and 95% CI of each variant was not changed significantly, and the pooled results of each grouped and subgroup analysis remained stable. Publication biases were not obvious from the funnel plots of all responsive variants (Supplementary Figure 2).

## Discussion

The current meta-analysis and systematic review is, as far as we are aware, the most comprehensive analysis of common *LRRK2* variants in PD to date, and revealed population heterogeneity to be a prominent factor in *LRRK2* allelic distribution.

Previous studies of ethnicity-specific *LRRK2* variation have focused primarily on the common variants G2019S, G2385R, and R1628P (Xie et al., 2014; Liu et al., 2016; Zhang et al., 2016; Zhao and Kong, 2016), showing that G2019S was more common in European and North American populations while G2385R and R1628P existed only in Asian populations (Guo et al., 2006). Our study replicated these results, and further demonstrated the importance of other variants, such as P755L, A419V, and R1398H. We demonstrated that G2019S, R1441C/G/H, A419V, G2385R, and R1628P, in descending order, carried the highest overall degrees of PD risk, as indicated by their ORs, which ranged from 13.16 to 1.83.

In East Asian populations, G2385R, R1628P, and A419V (arranged in descending order according to the frequency of their occurrence) were found to increase PD risk. Although previous studies have been quite successful at identifying high-frequency risk variants, our findings serve to highlight the fact that even those that occur at lower frequencies, such as A419V, should not be neglected, particularly in East Asian populations, even if clinically significant data may only be accessed in these cases through the use of larger sample sizes that are possible only with collaborative multi-center projects. We also determined that G2019S can increase PD risk in European/West Asian and mixed populations, while R1441C/G/H increases PD risk in European/West Asians only. The pooled analysis and frequency analysis of variants in *LRRK2* supported the differences in geographic distributions of *LRRK2* variants.

The 51-exon *LRRK2* gene has always posed a challenge for researchers interested in screening for the gene due to its large size. For the sake of improving the efficiency and economy of genetic diagnosis, it is thus of great importance to prioritize the identification of specific variations instead of sequencing the entire gene (Foroud, 2005). Based on calculations of the ORs associated with each of the *LRRK2* variants, we suggest that A419V, G2385R, and R1628P, and G2019S and R1441C/G/H should be screened for first in East Asians and European/West Asians, respectively. Variants with high OR *and* that occur at higher rates, such as G2385R in East Asian populations and G2019S in European/West Asian populations, should be, in particular, prioritized above all others.

**Figure 3:**
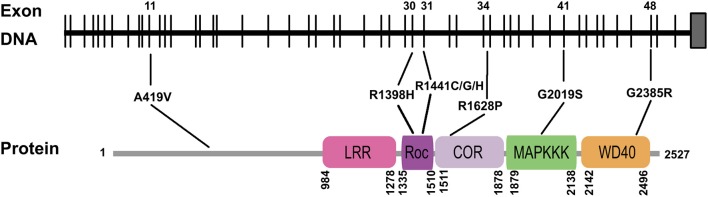
Schematic figure illustrating the distribution of mutations associated with various *LRRK2* variants demonstrated by meta-analysis to have a significant effect on PD risk. Only exons containing these mutations are shown. Note particular variants linked to mutations in functional domains. LRR, leucine rich repeat; ROC, Ras of complex proteins; COR, C-terminal of Roc; MAPKKK, kinase domain of MAPK; WD40, β-propeller.

Our meta-analysis also revealed additional details of how mutations affect *LRRK2* function, which could have potential ramifications with regards to our understanding of the mechanisms that underlie PD and the development of future treatment strategies. We determined, for instance, that all of the high-risk variants carried mutations in the exon regions of the *LRRK2* gene, and in the functional domains of the *LRRK2* protein (Figure 3). While the specific pathological mechanisms of these mutations are as yet unclear, it is possible that they can lead to an increase in the kinase activity of the protein, thereby increasing disease risk as well (West et al., 2005), as in the case of the G2019S “gain of function” feature (Luzon-Toro et al., 2007). If this is demonstrated to be true on a more general basis, it may be promising to target the variations in these vital regions for therapeutic purposes.

Certain *LRRK2* variants appear to be linked with clinical phenotypes. For instance, motor fluctuations were more frequent in the G2385R carriers than in non-carriers in PD patients. G2019S had better olfactory function and less likely to have depression than G2385R carriers (West et al., 2005). In a large meta-analysis of LRRK2 related clinical features by our group, we found that *LRRK2*-G2019S-related PD patients were likely to be female, had higher rates of early-onset PD and family history. Moreover, they tended to have high scores of Schwab & England, low Geriatric Depression Scale (GDS) scores, high University of Pennsylvania Smell Identification Test (UPSIT) scores and responded well to Levodopa. G2385R carriers tended to have family history, lower Hoehn and Yahr rating (H-Y) and higher Mini Mental State Examination (MMSE) scores. However, both G2019S and G2385R carriers were more likely to develop motor complications than non-carriers (Shu et al., 2018). Therefore, for the purposes of clinical genetic counseling and testing, the symptoms exhibited by the patient could be useful in guiding the decision of which *LRRK2* variant to screen for. Additionally, identifying the specific clinical features associated with carrier of particular *LRRK2* variants could also be useful for neurologists in prescribing the correct symptomatic treatments.

There are, of course, limitations to this study. Firstly, although we have endeavored to include every published case-control study in the meta-analysis, certain pieces of unpublished data, or articles that were written in languages other than English or Chinese may have been omitted unintentionally. Secondly, due to the lack of sufficient data, variants in populations other than East Asians and European/West Asians cannot be analyzed, and it is possible that biases may exist in our pooled analysis of different ethnic groups and in their demographic information, such as age and gender. Thirdly, stratified analysis can inadvertently increase the possibility of there being false positives, especially as the sample size is limited.

## Conclusion

In conclusion, we found that *LRRK2* variants A419V, G2019S, R1441C/G/H, G2385R, and R1628P were associated with increased PD risk while R1398H was associated with decreased risk. In East Asian populations, A419V, G2385R, and R1628P increased risk, while R1398H had the opposite effect. G2019S increased the risk in European/West Asian and mixed populations while R1441C/G/H increased the risk of PD in European/West Asians. Combined with frequency analysis, we suggest that A419V, G2385R, and R1628P should receive top priority for screening in East Asian populations and that a greater focus be placed on G2019S and R1441C/G/H in European/West Asian populations.

## Author Contributions

LS, YZ, and BT: chose the topic and designed the experiments; LS, YZ, and QS: performed the analysis; LS, YZ, QS, and BT: analyzed the data; LS, YZ, and BT: wrote the manuscript; HP: data management and figure modification.

### Conflict of Interest Statement

The authors declare that the research was conducted in the absence of any commercial or financial relationships that could be construed as a potential conflict of interest.
