# Supplementary material for: A Comprehensive Analysis of Population Differences in LRRK2 Variant Distribution in Parkinson's Disease
Source: Front Aging Neurosci. 2019 Jan 30;11:13. doi: 10.3389/fnagi.2019.00013 (PMC6363667; doi:10.3389/fnagi.2019.00013)
Supplement: Supplementary file 1 [file Table_1.DOCX]

Supplementary Material

A Comprehensive Analysis of Population Differences in *LRRK2* Variant Distribution in Parkinson’s disease

Li Shu^1 †^, Yuan Zhang^1 †^, Qiying Sun^2,3,4^, Hongxu Pan^1^, Beisha Tang^1, 3,4,5*^

**^†^** These authors have contributed equally to this work and are co-first authors.

^*^ Correspondence: Beisha Tang [bstang7398@163.com](mailto:bstang7398@163.com)

**Supplementary Table 1: The diagnostic criteria for PD patients and definition of control. Abbreviation: UKBB, UK PD Society Brain Bank Clinical Diagnostic Criteria; PD, Parkinson's disease.**

| Year | First author | Diagnostic criteria for PD patients | Definition of controls |
| --- | --- | --- | --- |
| 2005 | Tan EK | UKBB | without neurodegenerative diseases |
| 2005 | Nichols WC | UKBB | without PD |
| 2005 | Gilks WP | without description | without signs of parkinsonism |
| 2005 | Kachergus J | without description | without description |
| 2005 | Aasly JO | Gelb criteria | without movement disorders |
| 2005 | Mata IF | UKBB | without description |
| 2005 | Deng H | Gelb criteria | healthy |
| 2005 | Hernandez D | without description | without description |
| 2005 | Bras JM | UKBB | without PD |
| 2005 | Farrer M | Bower criteria | without PD |
| 2005 | Berg D | UKBB | without signs of parkinsonism |
| 2005 | Goldwurm S | UKBB | without PD |
| 2006 | Infante J | UKBB | without description |
| 2006 | Ozelius LJ | UKBB | without movement disorders |
| 2006 | Civitelli D | UKBB | healthy |
| 2006 | Williams-Gray CH | UKBB | without PD |
| 2006 | Di Fonzo A | UKBB | without PD |
| 2006 | Mata IF | UKBB | without description |
| 2006 | Carmine Belin A | UKBB | without neurological disease |
| 2006 | Marongiu R | UKBB | healthy |
| 2006 | Kay DM | UKBB | without neurological disease |
| 2006 | Pchelina SN | UKBB | healthy |
| 2006 | Schlitter AM | without description | healthy |
| 2006 | Clark LN | UKBB | without description |
| 2006 | Punia S | UKBB | without neurodegenerative diseases |
| 2006 | Wu T | UKBB | without neurological disease |
| 2006 | Deng H | Gelb criteria | without neurological disease |
| 2006 | Fung HC (1) | UKBB | healthy |
| 2006 | Fung HC (2) | Calne criteria | healthy |
| 2006 | Funayama M | UKBB | without description |
| 2007 | Tan EK(1) | UKBB | without neurodegenerative diseases |
| 2007 | Cossu G | UKBB | without PD |
| 2007 | Xiromerisiou G | Gelb criteria | without neurological disease |
| 2007 | Farrer MJ | Gelb criteria | without neurological disease |
| 2007 | Toft M | Gelb criteria | healthy |
| 2007 | Tan EK(2) | UKBB | without PD |
| 2007 | Orr-Urtreger A | UKBB | without movement disorders |
| 2007 | Li C | UKBB | healthy |
| 2008 | An XK | UKBB | without description |
| 2008 | Tan EK(1) | UKBB | healthy |
| 2008 | Ross OA | Gelb criteria | without neurological disease |
| 2008 | Aguiar Pde C | UKBB | healthy |
| 2008 | Kam D | UKBB | without signs of parkinsonism |
| 2008 | Hulihan MM | UKBB | without signs of parkinsonism |
| 2008 | Tan EK(2) | UKBB | without description |
| 2008 | Choi JM | UKBB | without neurological disease |
| 2008 | Lu CS | UKBB | healthy |
| 2008 | Patra B | UKBB | without signs of parkinsonism |
| 2008 | Tan EK(3) | UKBB | without neurodegenerative diseases |
| 2008 | Floris G | Gelb criteria | without signs of parkinsonism |
| 2008 | Tomiyama H | UKBB | without PD |
| 2008 | Bras J | Gelb criteria | healthy |
| 2009 | Gorostidi A | Gelb criteria | without description |
| 2009 | Zabetian CP | Calne criteria | without movement disorders |
| 2009 | Lesage S | UKBB | healthy |
| 2009 | Hassin-Baer S(a) | UKBB | healthy |
| 2009 | Hassin-Baer S(b) | UKBB | healthy |
| 2009 | Zhang Z | UKBB | without description |
| 2009 | Yu L | UKBB | without signs of parkinsonism |
| 2010 | Kim JM | UKBB | without signs of parkinsonism |
| 2010 | Miyake Y | UKBB | without neurodegenerative diseases |
| 2010 | Jasinska-Myga B | UKBB | healthy |
| 2010 | Yescas P | UKBB | without movement disorders |
| 2010 | Chen L | UKBB | without description |
| 2011 | Hu ZX | UKBB | without description |
| 2011 | Lin, C. H. | Gelb criteria | healthy |
| 2011 | Pulkes T | without description | without signs of parkinsonism |
| 2011 | Zheng Y | UKBB | healthy |
| 2011 | Yao LY | UKBB | without description |
| 2012 | Li NN | UKBB | healthy |
| 2012 | Vishwanathan Padmaja M | without description | without neurological disease |
| 2012 | Zhou Y | UKBB | without neurological disease |
| 2012 | Wang C | UKBB | healthy |
| 2012 | Yan H(1) | UKBB | without PD |
| 2012 | Yan H(2) | UKBB | healthy |
| 2013 | Fu X | UKBB | without description |
| 2013 | Wu-Chou YH | UKBB | healthy |
| 2013 | Cai J | UKBB | healthy |
| 2013 | Ma Q | UKBB | without description |
| 2013 | Gopalai AA | UKBB | without neurological disease |
| 2013 | Wu YR | Gelb criteria | healthy |
| 2013 | Li BF | UKBB | healthy |
| 2013 | Li ZM | UKBB | without description |
| 2014 | Chung SJ | UKBB | healthy |
| 2014 | Dan XJ | UKBB | healthy |
| 2014 | Pulkes T | UKBB | without signs of parkinsonism |
| 2014 | Chien HF | UKBB | without neurological disease |
| 2014 | Guo JF | UKBB | healthy |
| 2015 | Heckman MG(a) | Bower,Gelb criteria and UKBB | without movement disorders |
| 2015 | Heckman MG(b) | Bower,Gelb criteria and UKBB | without movement disorders |
| 2015 | Li K | UKBB | healthy |
| 2015 | Li XX | UKBB | without PD |
| 2015 | Duque AF | UKBB | without movement disorders |
| 2016 | Bandrés-Ciga S | UKBB | without neurological disease |
| 2017 | Landoulsi Z | UKBB | without neurological disease |
| 2018 | Emelyanov AK | UKBB | without description |
