# Supplementary material for: A Comprehensive Analysis of Population Differences in LRRK2 Variant Distribution in Parkinson's Disease
Source: Front Aging Neurosci. 2019 Jan 30;11:13. doi: 10.3389/fnagi.2019.00013 (PMC6363667; doi:10.3389/fnagi.2019.00013)
Supplement: Supplementary file 2 [file Table_2.DOCX]

Supplementary Material

A Comprehensive Analysis of Population Differences in *LRRK2* Variant Distribution in Parkinson’s disease

Li Shu^1 †^, Yuan Zhang^1 †^, Qiying Sun^2,3,4^, Hongxu Pan^1^, Beisha Tang^1, 3,4,5*^

**^†^** These authors have contributed equally to this work and are co-first authors.

^*^ Correspondence: Beisha Tang [bstang7398@163.com](mailto:bstang7398@163.com)

**Supplementary Table 3: Genotypes of 9 variants in *LRRK2* in all publications included. Abbreviation: PD, Parkinson’s disease. AA, wild type. Aa, heterozygous carriers. Aa, homozygous carriers.**

| Ethnicity | Country | Year and First author | Variants | Genotypes of PD | | | Genotypes of controls | | |
| --- | --- | --- | --- | --- | --- | --- | --- | --- | --- |
|  |  |  |  | AA | Aa | aa | AA | Aa | aa |
| East Asians | Taiwan | 2006 Di Fonzo A | A419V | 582 | 10 | 0 | 341 | 3 | 0 |
| East Asians | China | 2012 Li NN | A419V | 707 | 22 | 0 | 581 | 4 | 0 |
| East Asians | Malaysia | 2013 Gopalai AA | A419V | 403 | 1 | 0 | 421 | 3 | 0 |
| East Asians | China | 2015 Li K | A419V | 482 | 18 | 0 | 565 | 9 | 0 |
| African | Tunis | 2008 Hulihan MM | G2019S | 166 | 60 | 12 | 364 | 7 | 0 |
| African | Tunis | 2010 Jasinska-Myga B | G2019S | 167 | 61 | 12 | 365 | 7 | 0 |
| African | Tunis | 2017 Landoulsi Z | G2019S | 166 | 76 | 8 | 215 | 3 | 0 |
| East Asians | Singapore | 2005 Tan EK. | G2019S | 675 | 0 | 0 | 325 | 0 | 0 |
| East Asians | Taiwan | 2006 Fung HC(2) | G2019S | 343 | 0 | 0 | 213 | 0 | 0 |
| East Asians | India | 2006 Punia S | G2019S | 799 | 1 | 0 | 212 | 0 | 0 |
| East Asians | Japan | 2009 Zabetian CP | G2019S | 629 | 2 | 0 | 320 | 0 | 0 |
| East Asians | China | 2011Hu ZX | G2019S | 221 | 0 | 0 | 120 | 0 | 0 |
| East Asians | India | 2012 Vishwanathan Padmaja M | G2019S | 140 | 0 | 0 | 201 | 0 | 0 |
| East Asians | China | 2015 Li XX | G2019S | 312 | 0 | 0 | 360 | 0 | 0 |
| European/West Asians | Norway | 2005 Aasly JO | G2019S | 426 | 9 | 0 | 519 | 0 | 0 |
| European/West Asians | Germany | 2005 Berg D | G2019S | 336 | 1 | 0 | 1200 | 0 | 0 |
| European/West Asians | Portugal | 2005 Bras JM | G2019S | 117 | 7 | 0 | 126 | 0 | 0 |
| European/West Asians | UK | 2005 Gilks WP | G2019S | 474 | 8 | 0 | 345 | 0 | 0 |
| European/West Asians | Italy | 2005 Goldwurm S | G2019S | 616 | 13 | 0 | 440 | 0 | 0 |
| European/West Asians | America | 2005 Hernandez D | G2019S | 712 | 7 | 0 | 2680 | 0 | 0 |
| European/West Asians | Sweden | 2006 Carmine Belin A | G2019S | 280 | 4 | 0 | 304 | 1 | 0 |
| European/West Asians | Spain | 2006 Infante J | G2019S | 97 | 8 | 0 | 310 | 0 | 0 |
| European/West Asians | Italy | 2006 Marongiu R | G2019S | 1052 | 20 | 0 | 300 | 0 | 0 |
| European/West Asians | Spain | 2006 Mata IF | G2019S | 220 | 5 | 0 | 100 | 0 | 0 |
| European/West Asians | Russia | 2006 Pchelina SN | G2019S | 205 | 3 | 0 | 161 | 0 | 0 |
| European/West Asians | Germany | 2006 Schlitter AM | G2019S | 118 | 2 | 0 | 336 | 0 | 0 |
| European/West Asians | UK | 2006 Williams-Gray CH | G2019S | 517 | 2 | 0 | 887 | 0 | 0 |
| European/West Asians | Italy | 2007 Civitelli D | G2019S | 474 | 13 | 1 | 180 | 0 | 0 |
| European/West Asians | Italy | 2007 Cossu G | G2019S | 97 | 1 | 0 | 54 | 1 | 0 |
| European/West Asians | Israel | 2007 Orr-Urtreger A | G2019S | 414 | 58 | 0 | 1766 | 36 | 0 |
| European/West Asians | Greece | 2007 Xiromerisiou G | G2019S | 289 | 1 | 0 | 235 | 0 | 0 |
| European/West Asians | Portugal | 2008 Bras J | G2019S | 130 | 2 | 0 | 126 | 0 | 0 |
| European/West Asians | Italy | 2008 Floris G | G2019S | 350 | 6 | 0 | 208 | 0 | 0 |
| European/West Asians | America | 2008 Patra B | G2019S | 566 | 8 | 1 | 184 | 2 | 0 |
| European/West Asians | Spain | 2009 Gorostidi A | G2019S | 402 | 16 | 0 | 137 | 1 | 0 |
| European/West Asians | Israel | 2009 Hassin-Baer S | G2019S | 223 | 19 | 0 | 899 | 1 | 0 |
| European/West Asians | Spain | 2016 Bandrés-Ciga S | G2019S | 233 | 7 | 0 | 192 | 0 | 0 |
| European/West Asians | Russia | 2018 Emelyanov AK | G2019S | 750 | 12 | 0 | 400 | 0 | 0 |
| Hispanics | Brazil | 2008 Aguiar Pde C | G2019S | 68 | 4 | 0 | 72 | 0 | 0 |
| Hispanics | Mexico | 2010 Yescas P | G2019S | 318 | 1 | 0 | 200 | 0 | 0 |
| Hispanics | Colombian | 2015 Duque AF | G2019S | 152 | 2 | 0 | 161 | 1 | 0 |
| Mixed | America | 2005 Deng H | G2019S | 322 | 4 | 0 | 130 | 0 | 0 |
| Mixed | America | 2005 Farrer M | G2019S | 782 | 4 | 0 | 278 | 0 | 0 |
| Mixed | Europe and North America | 2005 Kachergus J | G2019S | 1041 | 13 | 0 | 2260 | 0 | 0 |
| Mixed | North America | 2005 Nichols WC | G2019S | 732 | 34 | 1 | 965 | 0 | 0 |
| Mixed | America | 2006 Clark LN | G2019S | 476 | 28 | 0 | 312 | 2 | 0 |
| Mixed | North America | 2006 Deng H | G2019S | 490 | 6 | 0 | 220 | 0 | 0 |
| Mixed | America | 2006 Kay DM | G2019S | 1498 | 19 | 1 | 1732 | 1 | 0 |
| Mixed | America | 2006 Ozelius LJ | G2019S | 98 | 22 | 0 | 313 | 4 | 0 |
| Mixed | Europe and North Africa | 2009 Lesage S | G2019S | 213 | 12 | 1 | 174 | 0 | 0 |
| Mixed | Multi-countries | 2014 Chien HF | G2019S | 100 | 0 | 0 | 100 | 0 | 0 |
| East Asians | Taiwan | 2006 Di Fonzo A | G2385R | 547 | 61 | 0 | 355 | 18 | 0 |
| East Asians | Japan | 2006 Funayama M | G2385R | 396 | 50 | 2 | 435 | 22 | 0 |
| East Asians | Taiwan | 2006 Fung HC(1) | G2385R | 278 | 27 | 0 | 175 | 1 | 0 |
| East Asians | Taiwan | 2007 Farrer MJ | G2385R | 376 | 34 | 0 | 322 | 13 | 0 |
| East Asians | China | 2007 Li C | G2385R | 221 | 14 | 0 | 214 | 0 | 0 |
| East Asians | Singapore | 2007 Tan EK(1) | G2385R | 458 | 36 | 1 | 476 | 18 | 0 |
| East Asians | Singapore | 2007 Tan EK(2) | G2385R | 164 | 2 | 0 | 304 | 2 | 0 |
| East Asians | China | 2008 An XK | G2385R | 529 | 70 | 1 | 323 | 11 | 0 |
| East Asians | Korea | 2008 Choi JM | G2385R | 63 | 9 | 0 | 95 | 5 | 0 |
| East Asians | Hong Kong | 2008 Kam D | G2385R | 75 | 6 | 1 | 31 | 0 | 0 |
| East Asians | Japan | 2009 Zabetian CP | G2385R | 560 | 69 | 2 | 1540 | 101 | 0 |
| East Asians | Korea | 2010 Kim JM | G2385R | 841 | 80 | 2 | 401 | 21 | 0 |
| East Asians | Japan | 2010 Miyake Y | G2385R | 199 | 30 | 0 | 335 | 23 | 0 |
| East Asians | Taiwan | 2011 Lin CH | G2385R | 419 | 32 | 1 | 275 | 13 | 0 |
| East Asians | Thailand | 2011 Pulkes T | G2385R | 153 | 1 | 0 | 156 | 0 | 0 |
| East Asians | China | 2012 Yan H(1) | G2385R | 333 | 21 | 0 | 333 | 7 | 0 |
| East Asians | China | 2012 Zhou Y | G2385R | 176 | 25 | 1 | 200 | 11 | 1 |
| East Asians | China | 2013 Cai J | G2385R | 461 | 49 | 0 | 538 | 12 | 0 |
| East Asians | China | 2013 Fu X | G2385R | 409 | 36 | 1 | 387 | 16 | 0 |
| East Asians | China | 2013 Li BF | G2385R | 178 | 19 | 0 | 197 | 5 | 0 |
| East Asians | China | 2013 Li ZM | G2385R | 213 | 24 | 0 | 186 | 4 | 0 |
| East Asians | China | 2013 Ma Q | G2385R | 213 | 24 | 0 | 186 | 4 | 0 |
| East Asians | Taiwan | 2013 Wu YR | G2385R | 520 | 53 | 0 | 482 | 21 | 0 |
| East Asians | Taiwan | 2013 Wu-Chou YH | G2385R | 863 | 78 | 0 | 581 | 37 | 0 |
| East Asians | Korean | 2014 Chung SJ | G2385R | 930 | 101 | 1 | 1139 | 61 | 1 |
| East Asians | China | 2014 Dan XJ | G2385R | 508 | 53 | 0 | 527 | 29 | 0 |
| East Asians | China | 2014 Guo JF | G2385R | 923 | 88 | 9 | 972 | 52 | 7 |
| East Asians | Taiwan | 2006 Di Fonzo A | P755L | 578 | 7 | 0 | 339 | 10 | 0 |
| East Asians | China | 2006 Wu T | P755L | 586 | 12 | 0 | 765 | 0 | 0 |
| East Asians | Singapore | 2008 Tan EK(1) | P755L | 200 | 4 | 0 | 229 | 6 | 0 |
| East Asians | Japan | 2008 Tomiyama H | P755L | 495 | 6 | 0 | 575 | 8 | 0 |
| East Asians | China | 2011 Yao LY | P755L | 397 | 4 | 0 | 396 | 2 | 0 |
| East Asians | Taiwan | 2013 Wu-Chou YH | P755L | 358 | 161 | 0 | 319 | 115 | 0 |
| East Asians | China | 2010 Chen L | R1398H | 361 | 68 | 1 | 364 | 87 | 1 |
| East Asians | Taiwan | 2013 Wu YR | R1398H | 483 | 86 | 4 | 407 | 95 | 1 |
| East Asians | Taiwan | 2013 Wu-Chou YH | R1398H | 433 | 83 | 3 | 354 | 77 | 3 |
| East Asians | Multi-countries | 2015 Heckman MG(a) | R1398H | 1084 | 238 | 23 | 708 | 221 | 9 |
| European/West Asians | Multi-countries | 2015 Heckman MG(b) | R1398H | 5182 | 682 | 30 | 3680 | 587 | 15 |
| European/West Asians | Italy | 2005 Goldwurm S | R1441C | 599 | 1 | 0 | 265 | 0 | 0 |
| European/West Asians | Italy | 2005 Mata IF | R1441G | 220 | 5 | 0 | 100 | 0 | 0 |
| European/West Asians | Spain | 2008 Floris G | R1441C | 354 | 2 | 0 | 208 | 0 | 0 |
| European/West Asians | Spain | 2009 Gorostidi A | R1441G | 363 | 55 | 0 | 138 | 0 | 0 |
| European/West Asians | Germany | 2005 Berg D | R793M | 335 | 2 | 0 | 1199 | 1 | 0 |
| European/West Asians | Norway | 2007 Toft M | R793M | 433 | 0 | 0 | 585 | 2 | 0 |
| European/West Asians | Spain | 2016 Bandrés-Ciga S | R793M | 239 | 1 | 0 | 192 | 0 | 0 |
| Mixed | America | 2005 Farrer M | R793M | 786 | 0 | 0 | 277 | 1 | 0 |
| East Asians | Taiwan | 2011 Lin CH | S1647T | 176 | 201 | 76 | 132 | 118 | 38 |
| East Asians | China | 2011 Zheng Y | S1647T | 162 | 145 | 99 | 167 | 184 | 61 |
| East Asians | China | 2012 Yan H(2) | S1647T | 140 | 175 | 39 | 70 | 78 | 12 |
| East Asians | Taiwan | 2013 Wu YR | S1647T | 232 | 266 | 75 | 183 | 250 | 70 |
