# Supplementary material for: A Comprehensive Analysis of Population Differences in LRRK2 Variant Distribution in Parkinson's Disease
Source: Front Aging Neurosci. 2019 Jan 30;11:13. doi: 10.3389/fnagi.2019.00013 (PMC6363667; doi:10.3389/fnagi.2019.00013)
Supplement: Supplementary file 3 [file Table_3.DOCX]

Supplementary Material

A Comprehensive Analysis of Population Differences in *LRRK2* Variant Distribution in Parkinson’s disease

Li Shu^1 †^, Yuan Zhang^1 †^, Qiying Sun^2,3,4^, Hongxu Pan^1^, Beisha Tang^1, 3,4,5*^

**^†^** These authors have contributed equally to this work and are co-first authors.

^*^ Correspondence: Beisha Tang [bstang7398@163.com](mailto:bstang7398@163.com)

**Supplementary Table 2**: **Genotype distributions and frequencies of *LRRK2* variants in different ethnic groups. Abbreviation: PD, Parkinson’s disease. AA, wild type. Aa, heterozygous carriers. Aa, homozygous carriers. GF, genotype frequency. MAF, minor allele frequency.**

| Variants | Groups | genotype% of PD | | | genotype% of Control | | | GF of PD | GF of Control | MAF of PD | MAF of Control |
| --- | --- | --- | --- | --- | --- | --- | --- | --- | --- | --- | --- |
|  |  | AA | Aa | aa | AA | Aa | aa |  |  |  |  |
| A419V | Africans | 0 | 0 | 0 | 0 | 0 | 0 | 0 | 0 | 0 | 0 |
|  | East Asians | 99.18% | 0.82% | 0.00% | 99.01% | 0.99% | 0.00% | 0.82% | 0.99% | 0.41% | 0.49% |
|  | European/West Asians | 0 | 0 | 0 | 0 | 0 | 0 | 0 | 0 | 0 | 0 |
|  | Hispanics | 0 | 0 | 0 | 0 | 0 | 0 | 0 | 0 | 0 | 0 |
|  | Mixed | 0 | 0 | 0 | 0 | 0 | 0 | 0 | 0 | 0 | 0 |
| P755L | Africans | 0 | 0 | 0 | 0 | 0 | 0 | 0 | 0 | 0 | 0 |
|  | East Asians | 93.09% | 6.91% | 0.00% | 94.90% | 5.10% | 0.00% | 6.91% | 5.10% | 3.45% | 2.55% |
|  | European/West Asians | 0 | 0 | 0 | 0 | 0 | 0 | 0 | 0 | 0 | 0 |
|  | Hispanics | 0 | 0 | 0 | 0 | 0 | 0 | 0 | 0 | 0 | 0 |
|  | Mixed | 0 | 0 | 0 | 0 | 0 | 0 | 0 | 0 | 0 | 0 |
| R793M | Africans | 0 | 0 | 0 | 0 | 0 | 0 | 0 | 0 | 0 | 0 |
|  | East Asians | 0 | 0 | 0 | 0 | 0 | 0 | 0 | 0 | 0 | 0 |
|  | European/West Asians | 99.70% | 0.30% | 0.00% | 99.85% | 0.15% | 0.00% | 0.30% | 0.15% | 0.15% | 0.08% |
|  | Hispanics | 0 | 0 | 0 | 0 | 0 | 0 | 0 | 0 | 0 | 0 |
|  | Mixed | 100.00% | 0.00% | 0.00% | 99.64% | 0.36% | 0.00% | 0.00% | 0.36% | 0.00% | 0.18% |
| R1398H | Africans | 0 | 0 | 0 | 0 | 0 | 0 | 0 | 0 | 0 | 0 |
|  | East Asians | 82.35% | 16.57% | 1.08% | 78.77% | 20.63% | 0.60% | 17.65% | 21.23% | 9.37% | 10.92% |
|  | European/West Asians | 87.92% | 11.57% | 0.51% | 85.94% | 13.71% | 0.35% | 12.08% | 14.06% | 6.29% | 7.20% |
|  | Hispanics | 0 | 0 | 0 | 0 | 0 | 0 | 0 | 0 | 0 | 0 |
|  | Mixed | 0 | 0 | 0 | 0 | 0 | 0 | 0 | 0 | 0 | 0 |
| R1441C/G/H | Africans | 0 | 0 | 0 | 0 | 0 | 0 | 0 | 0 | 0 | 0 |
|  | East Asians | 0 | 0 | 0 | 0 | 0 | 0 | 0 | 0 | 0 | 0 |
|  | European/West Asians | 96.06% | 3.94% | 0.00% | 100.00% | 0.00% | 0.00% | 3.94% | 0.00% | 1.97% | 0.00% |
|  | Hispanics | 0 | 0 | 0 | 0 | 0 | 0 | 0 | 0 | 0 | 0 |
|  | Mixed | 0 | 0 | 0 | 0 | 0 | 0 | 0 | 0 | 0 | 0 |
| R1628P | Africans | 0 | 0 | 0 | 0 | 0 | 0 | 0 | 0 | 0 | 0 |
|  | East Asians | 94.22% | 5.65% | 0.13% | 96.83% | 3.16% | 0.01% | 5.78% | 3.17% | 2.95% | 1.59% |
|  | European/West Asians | 0 | 0 | 0 | 0 | 0 | 0 | 0 | 0 | 0 | 0 |
|  | Hispanics | 0 | 0 | 0 | 0 | 0 | 0 | 0 | 0 | 0 | 0 |
|  | Mixed | 0 | 0 | 0 | 0 | 0 | 0 | 0 | 0 | 0 | 0 |
| S1647T | Africans | 0 | 0 | 0 | 0 | 0 | 0 | 0 | 0 | 0 | 0 |
|  | East Asians | 39.75% | 44.06% | 16.18% | 40.50% | 46.22% | 13.28% | 60.25% | 59.50% | 38.21% | 36.39% |
|  | European/West Asians | 0 | 0 | 0 | 0 | 0 | 0 | 0 | 0 | 0 | 0 |
|  | Hispanics | 0 | 0 | 0 | 0 | 0 | 0 | 0 | 0 | 0 | 0 |
|  | Mixed | 0 | 0 | 0 | 0 | 0 | 0 | 0 | 0 | 0 | 0 |
| G2019S | Africans | 68.54% | 27.06% | 4.40% | 98.23% | 1.77% | 0.00% | 31.46% | 1.77% | 17.93% | 0.88% |
|  | East Asians | 99.90% | 0.10% | 0.00% | 100.00% | 0.00% | 0.00% | 0.10% | 0.00% | 0.05% | 0.00% |
|  | European/West Asians | 97.49% | 2.49% | 0.02% | 99.65% | 0.35% | 0.00% | 2.51% | 0.35% | 1.26% | 0.17% |
|  | Hispanics | 98.72% | 1.28% | 0.00% | 99.77% | 0.23% | 0.00% | 1.28% | 0.23% | 0.64% | 0.12% |
|  | Mixed | 97.54% | 2.41% | 0.05% | 99.89% | 0.11% | 0.00% | 2.46% | 0.11% | 1.25% | 0.05% |
| G2385R | Africans | 0 | 0 | 0 | 0 | 0 | 0 | 0 | 0 | 0 | 0 |
|  | East Asians | 90.81% | 9.01% | 0.18% | 95.58% | 4.34% | 0.08% | 9.19% | 4.42% | 4.69% | 2.25% |
|  | European/West Asians | 0 | 0 | 0 | 0 | 0 | 0 | 0 | 0 | 0 | 0 |
|  | Hispanics | 0 | 0 | 0 | 0 | 0 | 0 | 0 | 0 | 0 | 0 |
|  | Mixed | 0 | 0 | 0 | 0 | 0 | 0 | 0 | 0 | 0 | 0 |
