# Supplementary material for: A Comprehensive Analysis of Population Differences in LRRK2 Variant Distribution in Parkinson's Disease
Source: Front Aging Neurosci. 2019 Jan 30;11:13. doi: 10.3389/fnagi.2019.00013 (PMC6363667; doi:10.3389/fnagi.2019.00013)
Supplement: Supplementary file 4 [file Data_Sheet_1.pdf]

Supplementary Material

A Comprehensive Analysis of Population Differences in *LRRK2* Variant Distribution in Parkinson's disease

Li Shu[1] †, Yuan Zhang[1] †, Qiying Sun[2,3,4], Hongxu Pan[1], Beisha Tang[1, 3,4,5*]

† These authors have contributed equally to this work and are co-first authors.

*** Correspondence: Beisha Tang bstang7398@163.com**

(A)

Study or Subgroup	Experimental Events	Experimental Total	Control Events	Control Total	Weight	Odds Ratio M-H, Fixed, 95% CI
**5.1.1 East Asians**						
2006Di Fonzo A	10	592	3	344	19.6%	1.95 [0.53, 7.15]
2012Li NN	22	729	4	585	22.6%	4.52 [1.55, 13.19]
2013Gopalai AA	1	404	3	424	15.3%	0.35 [0.04, 3.36]
2015Li K	18	500	9	574	42.4%	2.34 [1.04, 5.27]
**Subtotal (95% CI)**		**2225**		**1927**	**100.0%**	**2.45 [1.43, 4.20]**
Total events	51		19			
Heterogeneity: Chi² = 4.23, df = 3 (P = 0.24); I² = 29%						
Test for overall effect: Z = 3.28 (P = 0.001)						
**Total (95% CI)**		**2225**		**1927**	**100.0%**	**2.45 [1.43, 4.20]**
Total events	51		19			
Heterogeneity: Chi² = 4.23, df = 3 (P = 0.24); I² = 29%						
Test for overall effect: Z = 3.28 (P = 0.001)						
Test for subgroup differences: Not applicable						

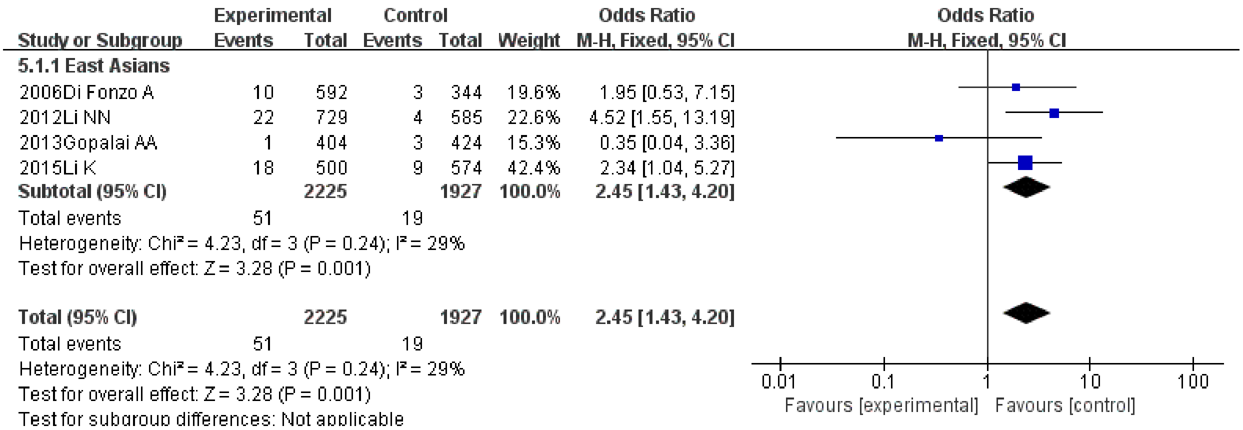

(B)

Study or Subgroup	Experimental Events	Experimental Total	Control Events	Control Total	Weight	Odds Ratio M-H, Fixed, 95% CI
**5.4.1 East Asians**						
2006Di Fonzo A	7	585	10	349	10.9%	0.41 [0.15, 1.09]
2006Wu T	12	598	0	765	0.4%	32.63 [1.93, 552.24]
2008Tan EK	4	204	6	235	4.8%	0.76 [0.21, 2.74]
2008Tomiyama H	6	501	8	583	6.4%	0.87 [0.30, 2.53]
2011Yao LY	4	401	2	398	1.7%	1.99 [0.36, 10.95]
2013Wu-Chou YH	161	519	115	434	75.8%	1.25 [0.94, 1.66]
**Subtotal (95% CI)**		**2808**		**2764**	**100.0%**	**1.24 [0.97, 1.59]**
Total events	194		141			
Heterogeneity: Chi² = 11.35, df = 5 (P = 0.04); I² = 56%						
Test for overall effect: Z = 1.71 (P = 0.09)						
**Total (95% CI)**		**2808**		**2764**	**100.0%**	**1.24 [0.97, 1.59]**
Total events	194		141			
Heterogeneity: Chi² = 11.35, df = 5 (P = 0.04); I² = 56%						
Test for overall effect: Z = 1.71 (P = 0.09)						
Test for subgroup differences: Not applicable						

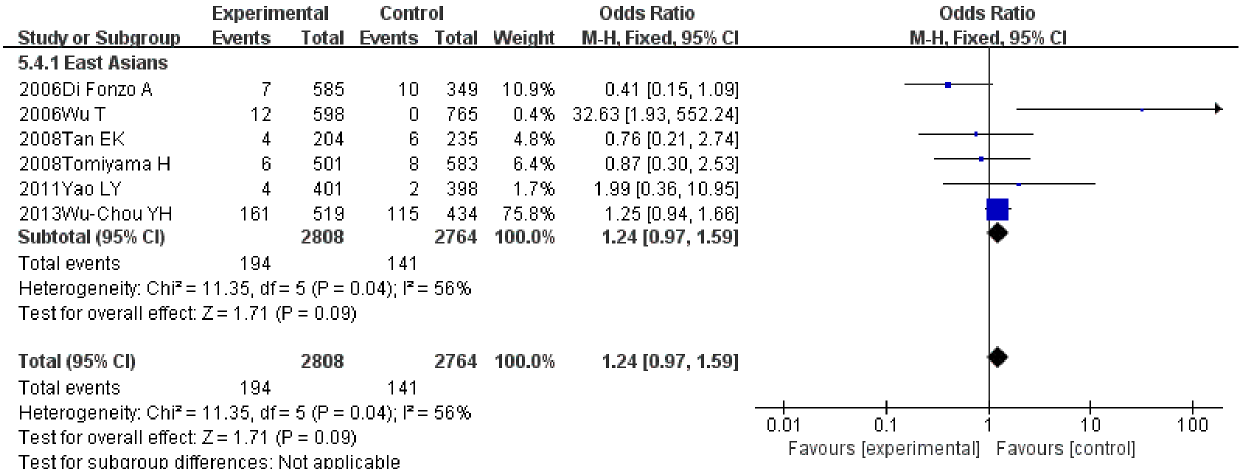

(C)

Study or Subgroup	Experimental Events	Experimental Total	Control Events	Control Total	Weight	Odds Ratio M-H, Fixed, 95% CI
**5.7.1 European/West Asians**						
2005Berg D	2	337	1	1200	8.2%	7.16 [0.65, 79.18]
2007Toft M	0	433	2	587	39.9%	0.27 [0.01, 5.64]
2016Bandrés-Ciga S	1	240	0	192	10.4%	2.41 [0.10, 59.52]
**Subtotal (95% CI)**		**1010**		**1979**	**58.4%**	**1.62 [0.41, 6.37]**
Total events	3		3			
Heterogeneity: Chi² = 2.86, df = 2 (P = 0.24); I² = 30%						
Test for overall effect: Z = 0.69 (P = 0.49)						
**5.7.2 Mixed**						
2005Farrer M	0	786	1	278	41.6%	0.12 [0.00, 2.90]
**Subtotal (95% CI)**		**786**		**278**	**41.6%**	**0.12 [0.00, 2.90]**
Total events	0		1			
Heterogeneity: Not applicable						
Test for overall effect: Z = 1.31 (P = 0.19)						
**Total (95% CI)**		**1796**		**2257**	**100.0%**	**0.99 [0.30, 3.31]**
Total events	3		4			
Heterogeneity: Chi² = 5.30, df = 3 (P = 0.15); I² = 43%						
Test for overall effect: Z = 0.01 (P = 0.99)						
Test for subgroup differences: Chi² = 2.17, df = 1 (P = 0.14), I² = 54.0%						

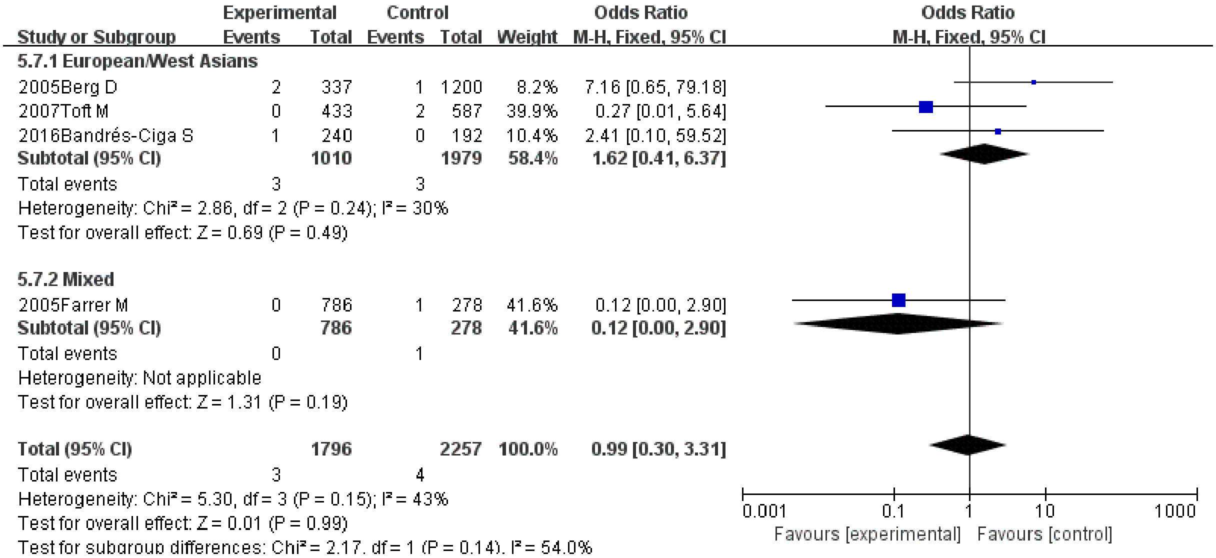

(D)

Study or Subgroup	Experimental Events	Experimental Total	Control Events	Control Total	Weight	Odds Ratio M-H, Fixed, 95% CI
**5.5.1 East Asians**						
2010Chen L	69	430	88	452	6.8%	0.79 [0.56, 1.12]
2013Wu YR	90	573	96	503	8.1%	0.79 [0.58, 1.08]
2013Wu-Chou YH	86	519	80	434	6.8%	0.88 [0.63, 1.23]
2015Heckman MG(a)	261	1345	230	938	20.6%	0.74 [0.61, 0.91]
**Subtotal (95% CI)**		**2867**		**2327**	**42.3%**	**0.78 [0.68, 0.90]**
Total events	506		494			
Heterogeneity: Chi² = 0.75, df = 3 (P = 0.86); I² = 0%						
Test for overall effect: Z = 3.50 (P = 0.0005)						
**5.5.2 European/West Asians**						
2015Heckman MG(b)	712	5894	602	4282	57.7%	0.84 [0.75, 0.94]
**Subtotal (95% CI)**		**5894**		**4282**	**57.7%**	**0.84 [0.75, 0.94]**
Total events	712		602			
Heterogeneity: Not applicable						
Test for overall effect: Z = 2.94 (P = 0.003)						
**Total (95% CI)**		**8761**		**6609**	**100.0%**	**0.81 [0.75, 0.89]**
Total events	1218		1096			
Heterogeneity: Chi² = 1.38, df = 4 (P = 0.85); I² = 0%						
Test for overall effect: Z = 4.50 (P < 0.00001)						
Test for subgroup differences: Chi² = 0.62, df = 1 (P = 0.43), I² = 0%						

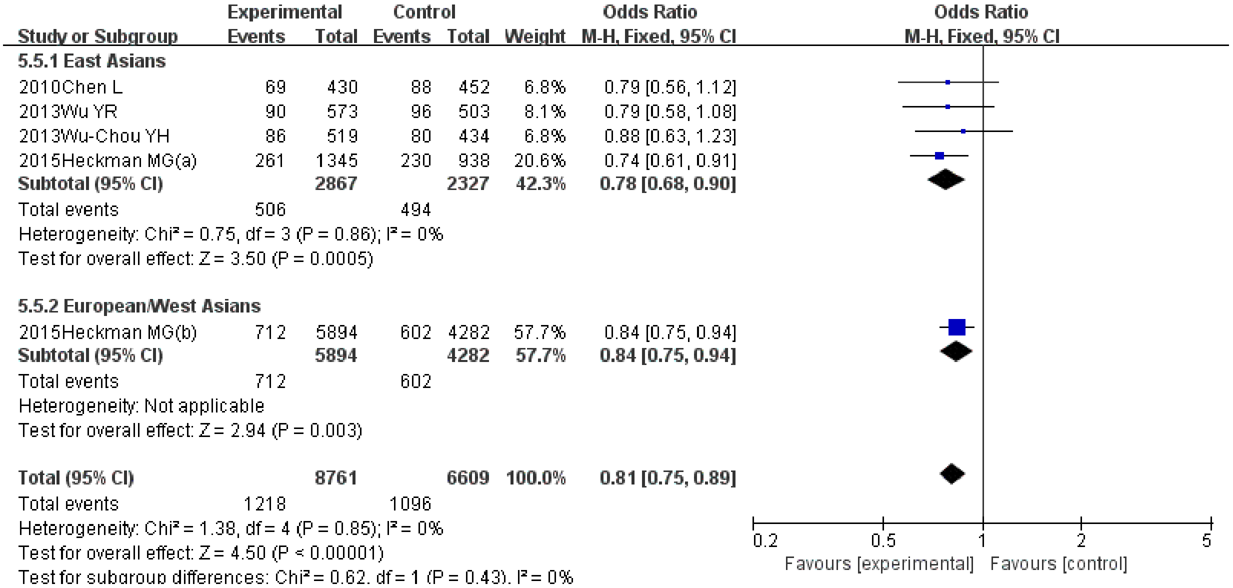

(E)

Study or Subgroup	Experimental Events	Experimental Total	Control Events	Control Total	Weight	Odds Ratio M-H, Fixed, 95% CI
**5.6.1 European/West Asians**						
2005Goldwurm S	1	600	0	265	26.2%	1.33 [0.05, 32.72]
2005Mata IF	2	356	0	208	23.7%	2.94 [0.14, 61.55]
2008Floris G	5	225	0	100	25.5%	5.01 [0.27, 91.54]
2009Gorostidi A	55	418	0	138	24.6%	42.29 [2.59, 689.36]
**Subtotal (95% CI)**		**1599**		**711**	**100.0%**	**12.75 [3.11, 52.27]**
Total events	63		0			
Heterogeneity: Chi² = 3.91, df = 3 (P = 0.27); I² = 23%						
Test for overall effect: Z = 3.53 (P = 0.0004)						
**Total (95% CI)**		**1599**		**711**	**100.0%**	**12.75 [3.11, 52.27]**
Total events	63		0			
Heterogeneity: Chi² = 3.91, df = 3 (P = 0.27); I² = 23%						
Test for overall effect: Z = 3.53 (P = 0.0004)						
Test for subgroup differences: Not applicable						

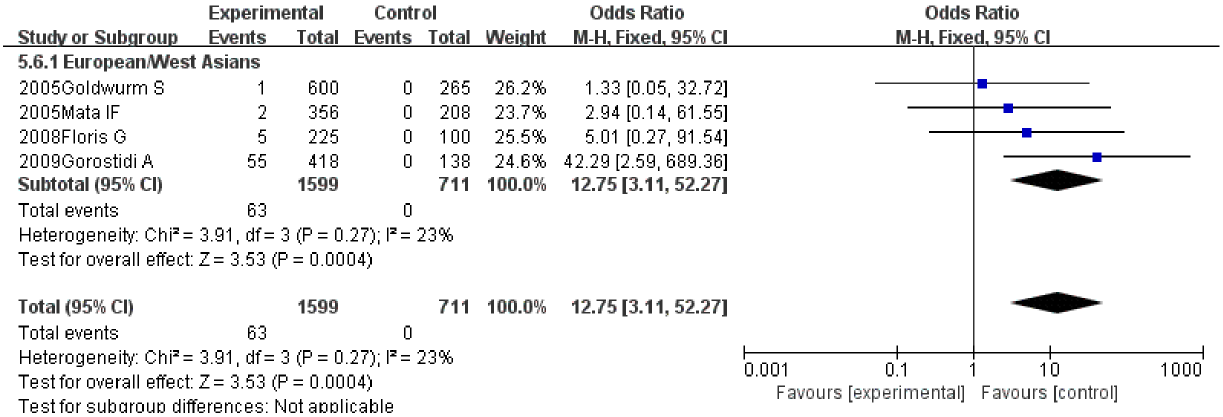

(F)

Study or Subgroup	Experimental Events	Experimental Total	Control Events	Control Total	Weight	Odds Ratio M-H, Fixed, 95% CI
**5.8.1 East Asians**						
2011Lin CH	277	453	156	291	20.6%	1.36 [1.01, 1.83]
2011Zheng Y	244	406	245	412	27.1%	1.03 [0.78, 1.36]
2012Yan H(2)	214	354	90	160	13.7%	1.19 [0.81, 1.74]
2013Wu YR	341	573	320	503	38.6%	0.84 [0.66, 1.08]
**Subtotal (95% CI)**		**1786**		**1366**	**100.0%**	**1.05 [0.91, 1.21]**
Total events	1076		811			
Heterogeneity: Chi² = 6.49, df = 3 (P = 0.09); I² = 54%						
Test for overall effect: Z = 0.61 (P = 0.54)						
**Total (95% CI)**		**1786**		**1366**	**100.0%**	**1.05 [0.91, 1.21]**
Total events	1076		811			
Heterogeneity: Chi² = 6.49, df = 3 (P = 0.09); I² = 54%						
Test for overall effect: Z = 0.61 (P = 0.54)						
Test for subgroup differences: Not applicable						

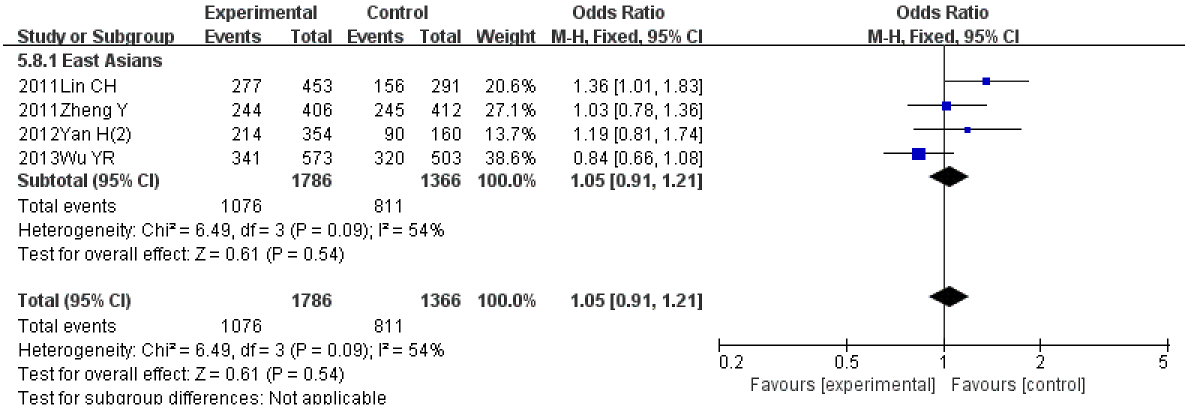

(G)

Study or Subgroup	Experimental Events	Experimental Total	Control Events	Control Total	Weight	Odds Ratio M-H, Fixed, 95% CI
**5.2.1 African**						
2008Hulihan MM	72	238	7	371	7.6%	22.55 [10.16, 50.06]
2010Jasinska-Myga B	73	240	7	372	7.6%	22.79 [10.27, 50.57]
2017Landoulsi Z	84	250	3	218	4.2%	36.27 [11.26, 116.76]
**Subtotal (95% CI)**		**728**		**961**	**19.4%**	**25.64 [15.38, 42.72]**
Total events	229		17			
Heterogeneity: Chi^2 = 0.52, df = 2 (P = 0.77); I^2 = 0%						
Test for overall effect: Z = 12.45 (P < 0.00001)						
**5.2.2 East Asians**						
2005Tan, E.K.	0	675	0	325		Not estimable
2006Fung HC(2)	0	343	0	213		Not estimable
2006Punia S	1	800	0	212	1.6%	0.80 [0.03, 19.64]
2009Zabetian CP	2	631	0	320	1.3%	2.55 [0.12, 53.18]
2011Hu ZX	0	221	0	120		Not estimable
2012Vishwanathan Padmaja M	0	140	0	201		Not estimable
2015Li XX	0	312	0	360		Not estimable
**Subtotal (95% CI)**		**3122**		**1751**	**2.9%**	**1.59 [0.18, 14.05]**
Total events	3		0			
Heterogeneity: Chi^2 = 0.27, df = 1 (P = 0.60); I^2 = 0%						
Test for overall effect: Z = 0.42 (P = 0.67)						
**5.2.3 European/West Asians**						
2005, 2008Bras JM	9	256	0	126	1.3%	9.71 [0.56, 168.20]
2005Aasly JO	9	435	0	519	0.9%	23.14 [1.34, 398.77]
2005Berg D	1	337	0	1200	0.4%	10.70 [0.44, 263.33]
2005Gilks WP	8	482	0	345	1.1%	12.38 [0.71, 215.18]
2005Goldwurm S	13	629	0	440	1.1%	19.29 [1.14, 325.38]
2005Hernandez D	7	719	0	2680	0.4%	56.43 [3.22, 989.23]
2006Carmine Belin A	4	284	1	305	1.9%	4.34 [0.48, 39.09]
2006Infante J	8	105	0	310	0.5%	54.14 [3.10, 946.50]
2006Marongjiu R	20	1072	0	300	1.5%	11.71 [0.71, 194.11]
2006Mata IF	5	225	0	100	1.3%	5.01 [0.27, 91.54]
2006Pchelina SN	3	208	0	161	1.1%	5.50 [0.28, 107.27]
2006Schlitter AM	2	120	0	336	0.5%	14.20 [0.68, 297.88]
2006Williams-Gray CH	2	519	0	887	0.7%	8.57 [0.41, 178.96]
2007Civitelli D	14	488	0	180	1.4%	11.03 [0.65, 185.89]
2007Cossu G	1	98	1	55	2.5%	0.56 [0.03, 9.08]
2007Orr-Urtreger A	58	472	36	1802	26.0%	6.87 [4.47, 10.56]
2007Xiromerisiou G	1	290	0	235	1.1%	2.44 [0.10, 60.18]
2008Floris G	6	356	0	208	1.2%	7.73 [0.43, 137.97]
2008Patra B	9	575	2	186	5.9%	1.46 [0.31, 6.83]
2009Gorostidi A	16	418	1	138	2.9%	5.45 [0.72, 41.50]
2009Hassin-Baer S	19	242	1	900	0.8%	76.60 [10.20, 575.23]
2016Bandrés-Ciga S	7	240	0	192	1.1%	12.37 [0.70, 217.90]
2018Emelyanov AK	12	762	0	400	1.3%	13.34 [0.79, 225.91]
**Subtotal (95% CI)**		**9332**		**12005**	**57.0%**	**8.71 [6.12, 12.38]**
Total events	234		42			
Heterogeneity: Chi^2 = 20.29, df = 22 (P = 0.56); I^2 = 0%						
Test for overall effect: Z = 12.05 (P < 0.00001)						
**5.2.4 Hispanicas**						
2008Aguiar Pde C	4	72	0	72	0.9%	9.53 [0.50, 180.25]
2010Yescas P	1	319	0	200	1.2%	1.89 [0.08, 46.59]
2015Duque AF	2	154	1	162	1.9%	2.12 [0.19, 23.60]
**Subtotal (95% CI)**		**545**		**434**	**4.1%**	**3.75 [0.80, 17.69]**
Total events	7		1			
Heterogeneity: Chi^2 = 0.78, df = 2 (P = 0.68); I^2 = 0%						
Test for overall effect: Z = 1.67 (P = 0.09)						
**5.2.5 Mixed**						
2005Deng H	4	326	0	130	1.4%	3.64 [0.19, 68.12]
2005Farrer M	4	786	0	278	1.5%	3.20 [0.17, 59.69]
2005Kachergus J	13	1054	0	2260	0.6%	58.60 [3.48, 986.72]
2005Nichols WC	35	767	0	965	0.8%	93.58 [5.73, 1528.09]
2006Clark LN	28	504	2	314	4.6%	9.18 [2.17, 38.79]
2006Deng H	6	496	0	220	1.4%	5.84 [0.33, 104.19]
2006Kay DM	20	1518	1	1733	1.8%	23.12 [3.10, 172.51]
2006Ozelius LJ	22	120	4	317	3.6%	17.57 [5.91, 52.21]
2009Lesage S	13	226	0	174	1.1%	22.07 [1.30, 373.86]
2014Chien HF	0	100	0	100		Not estimable
**Subtotal (95% CI)**		**5897**		**6491**	**16.7%**	**18.12 [9.26, 35.44]**
Total events	145		7			
Heterogeneity: Chi^2 = 6.02, df = 8 (P = 0.65); I^2 = 0%						
Test for overall effect: Z = 8.46 (P < 0.00001)						
**Total (95% CI)**		**19624**		**21642**	**100.0%**	**13.16 [10.16, 17.04]**
Total events	618		67			
Heterogeneity: Chi^2 = 50.46, df = 39 (P = 0.10); I^2 = 23%						
Test for overall effect: Z = 19.54 (P < 0.00001)						
Test for subgroup differences: Chi^2 = 18.56, df = 4 (P = 0.0010), I^2 = 78.4%						

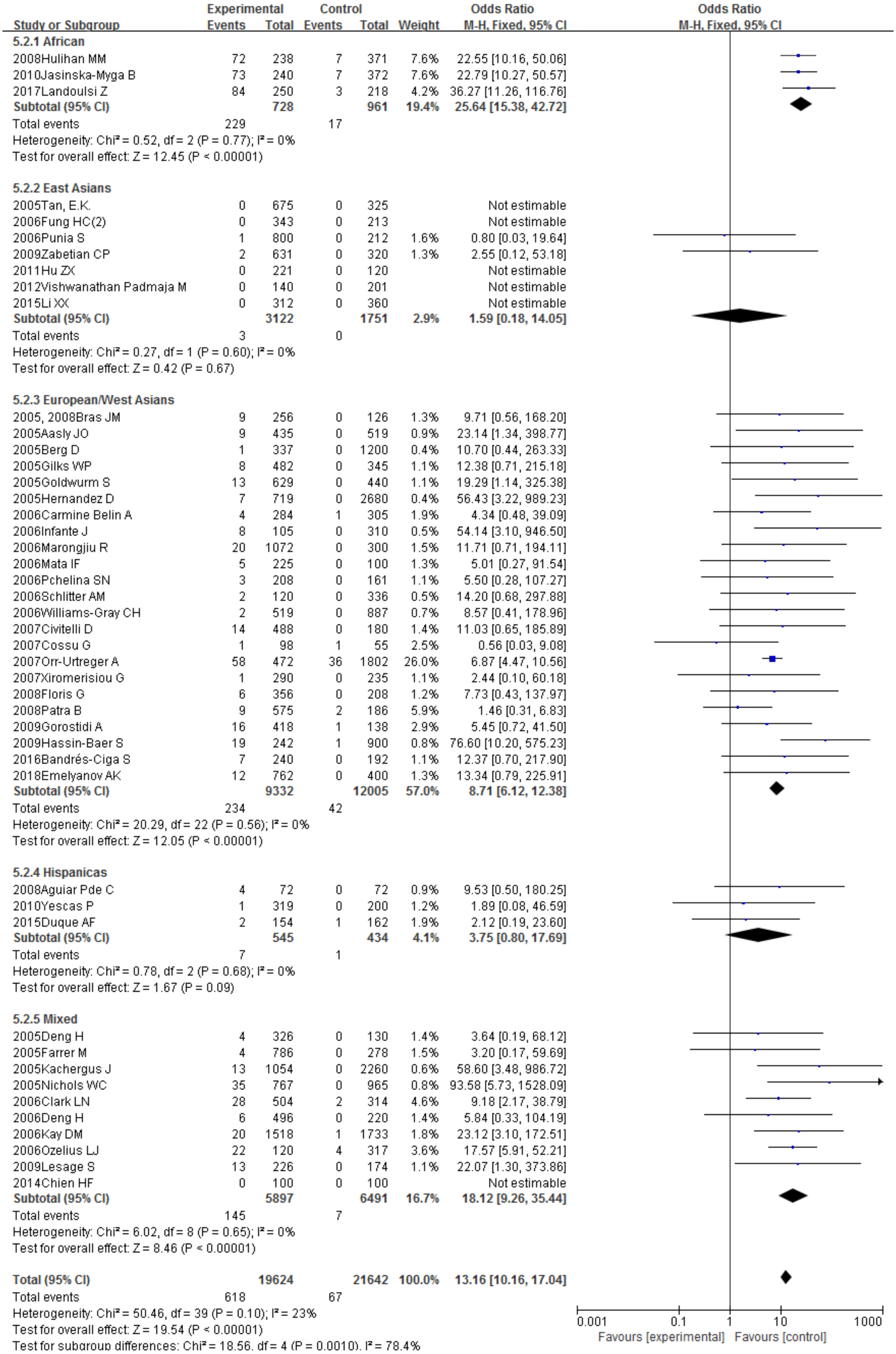

(H)

Study or Subgroup	Experimental Events	Experimental Total	Control Events	Control Total	Weight	Odds Ratio M-H, Fixed, 95% CI
**5.3.1 East Asians**						
2006Di Fonzo A	61	608	18	373	4.5%	2.20 [1.28, 3.78]
2006Funayama M	52	448	22	457	4.3%	2.60 [1.55, 4.35]
2006Fung HC(1)	27	305	1	176	0.3%	17.00 [2.29, 126.20]
2007Farrer MJ	34	410	13	335	3.0%	2.24 [1.16, 4.32]
2007Li C	14	235	0	214	0.1%	28.08 [1.66, 473.72]
2007Tan EK(1)	37	495	18	494	3.8%	2.14 [1.20, 3.81]
2007Tan EK(2)	2	166	2	306	0.3%	1.85 [0.26, 13.28]
2008An XK	71	600	11	334	2.8%	3.94 [2.06, 7.55]
2008Choi JM	9	72	5	100	0.8%	2.71 [0.87, 8.48]
2008Kam D	7	82	0	31	0.1%	6.26 [0.35, 112.92]
2009Zabetian CP	71	631	101	1641	11.2%	1.93 [1.41, 2.66]
2010Kim JM	82	923	21	422	5.9%	1.86 [1.14, 3.05]
2010Miyake Y	30	229	23	358	3.5%	2.20 [1.24, 3.89]
2011Lin CH	33	453	13	291	3.3%	1.68 [0.87, 3.25]
2011Pulkes T	1	154	0	156	0.1%	3.06 [0.12, 75.66]
2012Yan H(1)	21	354	7	340	1.5%	3.00 [1.26, 7.15]
2012Zhou Y	26	202	12	212	2.3%	2.46 [1.21, 5.02]
2013Cai J	49	510	12	550	2.4%	4.77 [2.50, 9.07]
2013Fu X	37	446	16	403	3.5%	2.19 [1.20, 4.00]
2013Li BF	19	197	5	202	1.0%	4.21 [1.54, 11.50]
2013Li ZM	24	237	4	190	0.9%	5.24 [1.79, 15.38]
2013Ma Q	24	237	4	190	0.9%	5.24 [1.79, 15.38]
2013Wu YR	53	573	21	503	4.6%	2.34 [1.39, 3.94]
2013Wu-Chou YH	78	941	37	618	9.2%	1.42 [0.95, 2.13]
2014Chung SJ	102	1032	62	1201	11.6%	2.01 [1.45, 2.80]
2014Dan XJ	53	561	29	556	5.9%	1.90 [1.19, 3.03]
2014Guo JF	97	1020	59	1031	12.0%	1.73 [1.24, 2.42]
**Subtotal (95% CI)**		**12121**		**11684**	**100.0%**	**2.27 [2.03, 2.53]**
Total events	1114		516			
Heterogeneity: Chi² = 33.40, df = 26 (P = 0.15); I² = 22%						
Test for overall effect: Z = 14.46 (P < 0.00001)						
**Total (95% CI)**		**12121**		**11684**	**100.0%**	**2.27 [2.03, 2.53]**
Total events	1114		516			
Heterogeneity: Chi² = 33.40, df = 26 (P = 0.15); I² = 22%						
Test for overall effect: Z = 14.46 (P < 0.00001)						
Test for subgroup differences: Not applicable						

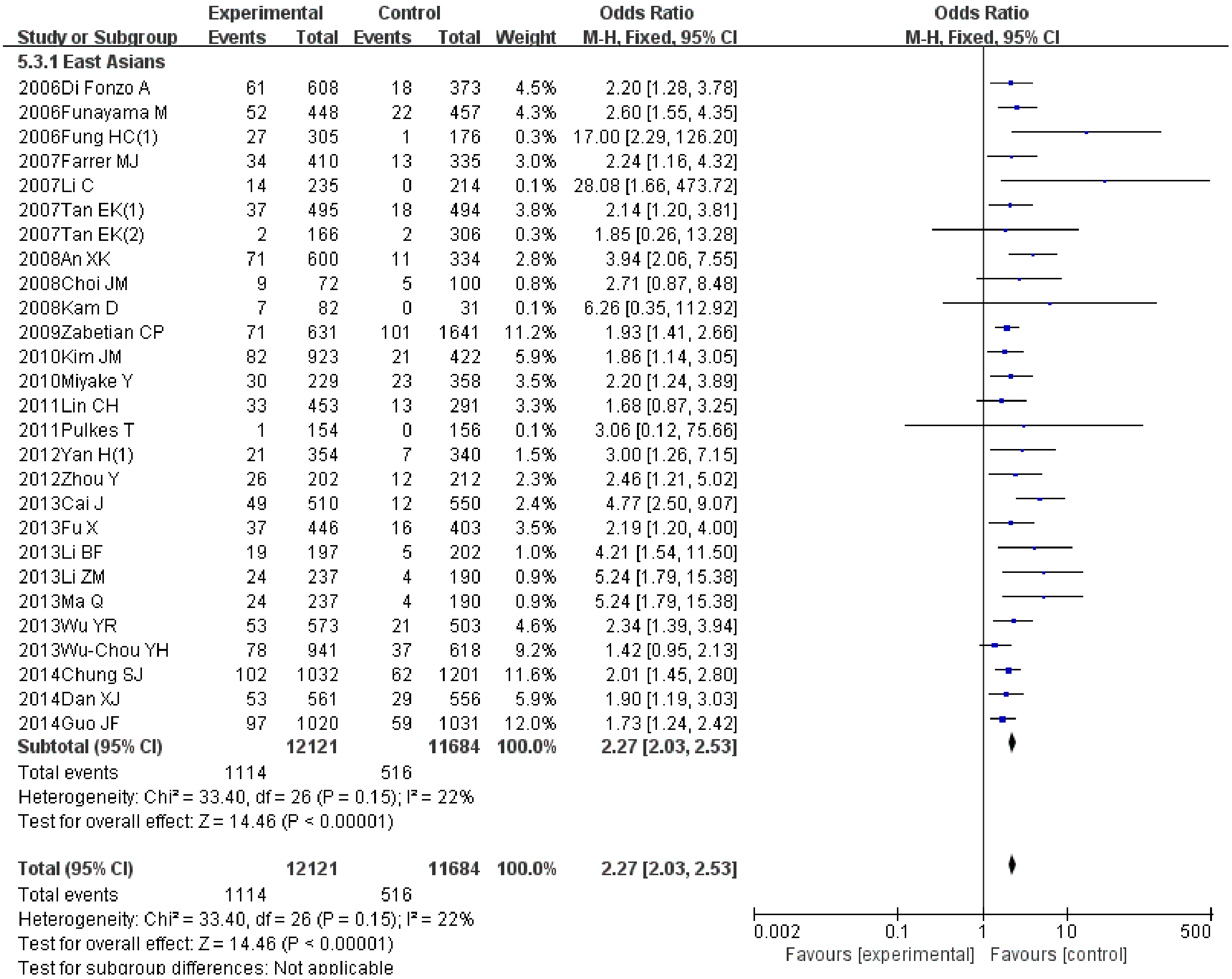

Supplementary Figure 1(A-H). Forest plots of the association between *LRRK2* variants and PD risks in total population and by ethnicity. (A)-(H) represented the pooled results of A419V, P755L, R793M, R1398H, R1441C/G/H, S1647T, G2019S, G2385R variants separately.
