# Supplementary material for: A Comprehensive Analysis of Population Differences in LRRK2 Variant Distribution in Parkinson's Disease
Source: Front Aging Neurosci. 2019 Jan 30;11:13. doi: 10.3389/fnagi.2019.00013 (PMC6363667; doi:10.3389/fnagi.2019.00013)
Supplement: Supplementary file 5 [file Data_Sheet_2.pdf]

Supplementary Material

A Comprehensive Analysis of Population Differences in *LRRK2* Variant Distribution in Parkinson's disease

Li Shu[1] †, Yuan Zhang[1] †, Qiying Sun[2,3,4], Hongxu Pan[1], Beisha Tang[1, 3,4,5*]

† These authors have contributed equally to this work and are co-first authors.

*** Correspondence: Beisha Tang bstang7398@163.com**

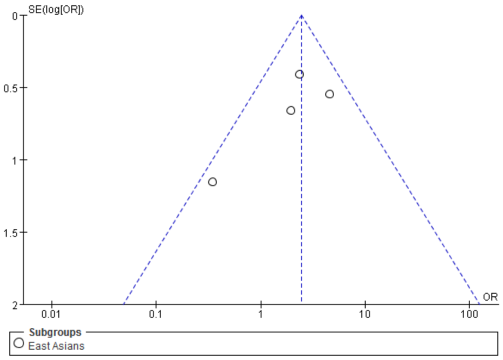

(A)

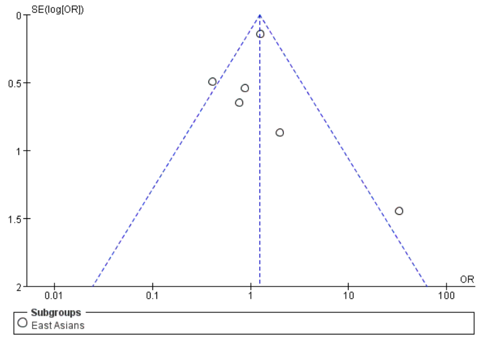

(B)

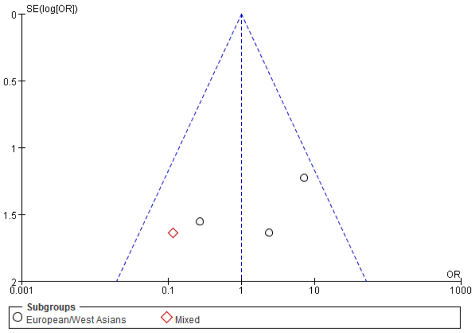

(C)

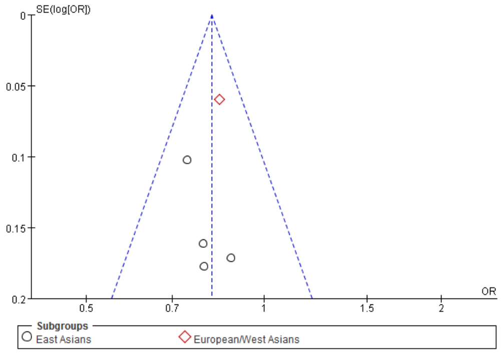

(D)

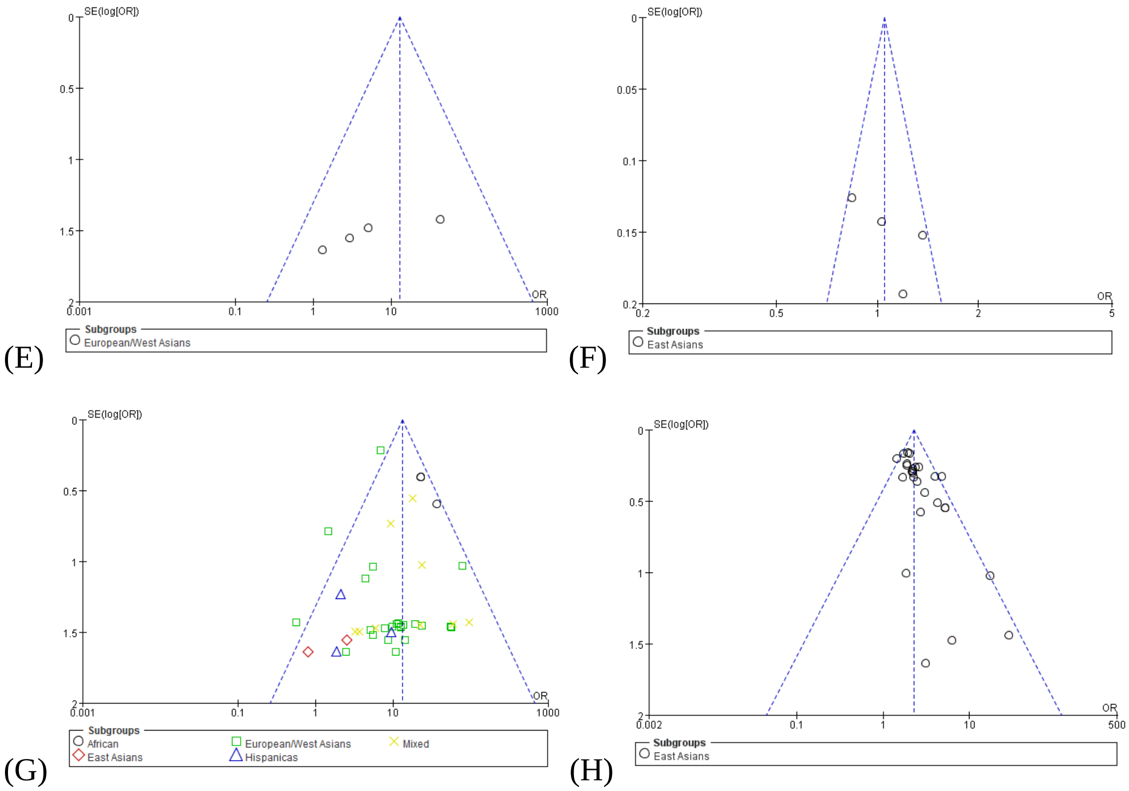

Supplementary Figure 2: Funnel plots of the association between *LRRK2* variants and PD risks in total population and by ethnicity. (A)-(H) represented the pooled results of A419V, P755L, R793M, R1398H, R1441C/G/H, S1647T, G2019S, G2385R variants separately.
